# Deficiencies in Jasmonate-Mediated Plant Defense Reveal Quantitative Variation in *Botrytis cinerea* Pathogenesis

**DOI:** 10.1371/journal.ppat.1000861

**Published:** 2010-04-15

**Authors:** Heather C. Rowe, Justin W. Walley, Jason Corwin, Eva K.-F. Chan, Katayoon Dehesh, Daniel J. Kliebenstein

**Affiliations:** 1 Department of Plant Sciences, University of California Davis, Davis, California, United States of America; 2 Department of Plant Biology, University of California Davis, Davis, California, United States of America; The University of North Carolina at Chapel Hill, United States of America

## Abstract

Despite the described central role of jasmonate signaling in plant defense against necrotrophic pathogens, the existence of intraspecific variation in pathogen capacity to activate or evade plant jasmonate-mediated defenses is rarely considered. Experimental infection of jasmonate-deficient and jasmonate-insensitive *Arabidopsis thaliana* with diverse isolates of the necrotrophic fungal pathogen *Botrytis cinerea* revealed pathogen variation for virulence inhibition by jasmonate-mediated plant defenses and induction of plant defense metabolites. Comparison of the transcriptional effects of infection by two distinct *B. cinerea* isolates showed only minor differences in transcriptional responses of wild-type plants, but notable isolate-specific transcript differences in jasmonate-insensitive plants. These transcriptional differences suggest *B. cinerea* activation of plant defenses that require plant jasmonate signaling for activity in response to only one of the two *B. cinerea* isolates tested. Thus, similar infection phenotypes observed in wild-type plants result from different signaling interactions with the plant that are likely integrated by jasmonate signaling.

## Author Summary

While many important elements of plant defense signaling have been identified, the function of these defense signaling pathways may mask additional variation in the plant–pathogen interaction, including both pathogen variation and variation in downstream plant defense responses. Jasmonate plant hormones contribute to both plant development and defense, including plant defense against necrotrophic fungal pathogens such as the grey mold *Botrytis cinerea*. Ten diverse *B. cinerea* isolates all showed increased virulence and decreased induction of a plant antimicrobial metabolite in experimental infections of *Arabidopsis thaliana* lacking functional jasmonate signaling. Yet within this consistent result, *B. cinerea* isolates varied considerably. Through comparing the transcript profiles of *A. thaliana* infected with the two most disparate *B. cinerea* isolates, we found that wild-type plants showed similar transcriptional responses to infection with these two isolates, but the absence of functional jasmonate signaling revealed dramatic differences in plant response, including groups of co-regulated genes that may participate in undescribed plant response networks. Jasmonate signaling appears to integrate plant responses to diverse pathogen inputs, and its absence may reveal novel aspects of plant–pathogen interaction.

## Introduction

Jasmonate-mediated signaling controls diverse aspects of plant growth and defense. In particular, jasmonate signaling exerts a major influence on plant response to wounding, chewing insects, and necrotrophic pathogens such as *Botrytis cinerea*, *Alternaria brassicicola*, *Plectosphaerella cucumerina*, and *Sclerotinia sclerotiorum* [1]–[6]. Appropriate plant responses to these diverse stimuli are believed to be tailored by cross-talk between jasmonate and other hormone signals, such as salicylic acid (SA), ethylene, and abscisic acid (ABA) [7]–[14]. Jasmonate signaling therefore does not mediate plant defense in isolation, but as part of a network of signals with the potential for positive and negative interactions. These signals include inputs from the pathogen that may influence the plant's defense response with positive or negative outcomes for the plant.

Two major pathogen classes are roughly delineated by the pathogen's “lifestyle”: biotrophic pathogens infect living host cells and necrotrophic pathogens kill cells prior to consuming them [15]–[17]. This difference in the pathogen's mode of attack strongly influences which signaling networks mediate the plant response. Plant responses to biotrophic pathogens are largely mediated by salicylate signaling with an emphasis on specific recognition of pathogen effectors by the products of plant resistance (R) genes, often characterized by nucleotide binding sites and leucine-rich repeats [18], [19]. Plant responses to necrotrophic pathogens appear to be mediated by a complex web of signaling dominated by jasmonates and ethylene [20]–[22]. Specific recognition of necrotrophic pathogens by the products of plant R genes is currently unknown, although recent identification of a gene possessing structural similarities to R-genes as the molecular basis of a quantitative trait locus (QTL) affecting resistance of *Arabidopsis thaliana* to multiple necrotrophic and hemibiotrophic pathogens has been suggested to link mechanisms of defense against biotrophic and necrotrophic pathogens [23]. While plants respond to biotrophic and necrotrophic pathogens via different signaling systems, these systems activate common defense responses, such as the production of the *A. thaliana* defense metabolite, camalexin. Thus, common responses may be controlled by distinct regulatory networks.

The simplified statement that biotrophic and necrotrophic pathogens activate distinct, but overlapping, defense signaling pathways is largely based on observation of single genotypes of the respective pathogens. Yet biotrophic pathogen species exhibit considerable variation in activation of plant defense signaling. This biotroph variation is largely associated with diversity in the R-gene mediated specificity of plant-pathogen recognition, a phenomenon not documented for necrotrophic pathogens [24]–[26]. Examples of naturally occurring intraspecific pathogen variation affecting plant defense against necrotrophs include variation in toxin production by pathogens and variation in pathogen tolerance or detoxification of plant-produced defense compounds [27]–[31].

While activating plant defense signaling should logically hinder infection, pathogens may manipulate plant defense signaling to improve pathogenesis by diverting plant resources toward defense strategies that are less effective against, or actually increase sensitivity to, the pathogen. Pathogens are known to produce plant hormones or analogues such as coronatine, gibberellins or ABA, and the production of these compounds has been associated with virulence [32]–[34]. Interestingly, the ability to produce these compounds may vary among isolates of the same pathogen species as shown by a survey of 95 strains of *Pseudomonas syringae* where only 15% assayed positively for coronatine production [35]. While production of ABA by pathogenic fungi has not been as extensively assayed, ABA-overproducing and ABA-deficient *B. cinerea* strains have been described [36]. In addition, some *B. cinerea* isolates produce ethylene [37]. Thus, while elements of plant defense signaling may be associated with resistance to particular pathogens, pathogen variation in activation, manipulation, and response to plant defense signaling may alter these associations. Despite available literature suggesting that *B. cinerea* natural diversity could impact plant defense signaling, this diversity has not been routinely integrated into studies of plant—pathogen interaction.

Unlike many pathogens that possess shorter or longer biotrophic stages, *B. cinerea* is identified as an unambiguously necrotrophic pathogen [17], [20]. This ascomycete fungus occupies broad geographic and host ranges and exhibits a high degree of genetic and phenotypic variability [38]–[40]. However, this variation has been little explored in the context of plant defense signaling. Testing the interaction between a collection of *B. cinerea* isolates and *A. thaliana* mutant genotypes with defined deficiencies in jasmonate signaling revealed significant variation in plant response to *B. cinerea* isolates that was not apparent in wild-type plants. This included variation in lesion phenotype, altered mRNA transcript accumulation responses, and variation in accumulation of the *A. thaliana* defense metabolite camalexin. An unexpected dependency of camalexin accumulation in response to *B. cinerea* infection on intact jasmonate signaling was also revealed. The results presented here, while not contradicting the accepted view that jasmonate-mediated defense is vital for plant resistance to *B. cinerea*, suggest that additional pathways modulate *A. thaliana*—*B. cinerea* interactions. Finally, the architecture of plant defense signaling networks that provide resistance to necrotrophic pathogens is not static, and will vary with the pathogen genotype investigated.

## Results

### Pathogen variation in jasmonate-dependent infection phenotypes

**Figure 1:**
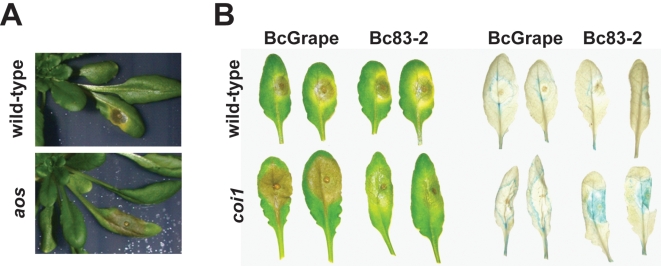
*A. thaliana* leaves showing necrotic lesions formed by *B. cinerea* infection at 72hpi. Horizontal labels indicate the *B. cinerea* isolate used for inoculum. Vertical labels show plant genotypes. **A**) BcGrape lesions on wild-type and *aos* plants; **B**) lesions on wild-type and *coi1* detached leaves (left); ProCYP79B2:GUS, COI1 (WT) and *coi1* leaves additionally containing a transgenically-introduced fusion of the CYP79B2 promoter region to a GUS (*uidA*) reporter infected with BcGrape or Bc83-2 and subsequently stained for the presence of GUS activity (right).

To test effects of jasmonate-mediated plant defense on diverse *B. cinerea* isolates, *A. thaliana* leaves of the *aos* genotype (deficient in jasmonate biosynthesis) and its corresponding wild-type were inoculated with 10 diverse *B. cinerea* isolates, two abiotic elicitors (acifluorfen and AgNO_3_), or a mock inoculation (Table 1) [41]. Visible initiation of leaf necrotic lesions was observed between 24 and 48 hours post inoculation with *B. cinerea*. While tissue necrosis of *aos* plants initiated within a time frame similar to wild-type plants, lesions expanded more rapidly in *aos* plants, with near total consumption of the leaf by *B. cinerea* between 72 and 96hpi. *aos* mutant leaves failed to develop the zone of chlorosis surrounding the developing lesion that is often observed in *B. cinerea* infections (Figure 1A).

**Table 1:** *B. cinerea* isolates and abiotic treatments.

Treatment	Source	Host	References
*(B. cinerea)*			
BMM	O. Lamotte, University of Fribourg	geranium	[86], [102]
FDOR2	isolated 2005, Watsonville CA	raspberry	[89]
FRESA	isolated 2005, San Diego CA	strawberry	[103]
GRAPE	M. Vivier, University of Capetown	grape	[89], [103], [104]
KB2	D. Gubler, University of California Davis	grape	[89], [94]
DN	isolated 2005, Davis CA	citrus	[89], [103]
PEPPER	K. Denby, University of Warwick	pepper	[89], [104]
83-2	D. Margosan, USDA, Parlier CA	rose	[89]
RASP	isolated 2005, Watsonville CA	raspberry	[89]
SUPER	isolated 2006, Davis CA	tomato	
*(abiotic)*			
Acifluorfen	Sigma-Aldrich, St. Louis MO	NA	[104]
AgNO_3_	Sigma-Aldrich, St. Louis MO	NA	[105]

The name and source of all *B. cinerea* isolates used in this study are provided, as well as collection host and published references to the isolate (if available). Commercial source and published references are provided for compounds used as abiotic elicitors.

**Figure 2:**
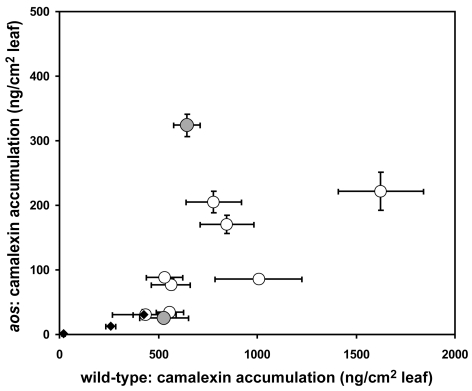
Variation in camalexin accumulation in jasmonate-deficient *A. thaliana*. Mean (± SE) camalexin accumulation in detached leaves of wild-type and jasmonate-deficient (*aos*) *A. thaliana* treated with 10 different isolates of *B. cinerea* (circles) or abiotic treatments (mock inoculation, AgNO_3_, and acifluorfen) (diamonds). 10 leaves per genotype×isolate combination were measured. Vertical error bars not visible are contained within the boundary of the data point marker. Filled circles highlight *B. cinerea* isolates selected as inducing high (Bc83-2) and low (BcGrape) relative levels of camalexin accumulation in *aos* plants.

A comparison of camalexin accumulation in wild-type versus *aos* leaves induced by 10 *B. cinerea* isolates revealed significant diversity (Figure 2). Among the *B. cinerea* isolate treatments tested, camalexin accumulation in *aos* leaves ranged from 5% to 50% of camalexin accumulation in wild-type leaves, with a median camalexin accumulation among *B. cinerea* infections of 14% wild-type levels. Mock treatment, acifluorfen, and AgNO_3_ induced camalexin in *aos* leaves at 5–7% wild-type levels. In no case was the absence of jasmonate synthesis associated with increased camalexin accumulation. To explore the observed pathogen variation in interaction with jasmonate-deficient genotypes and activation of metabolic defense, the two *B. cinerea* isolates inducing camalexin accumulation in the *aos* leaves at the highest and lowest levels relative to wild-type leaves, BcGrape (5%) and Bc83-2 (50%) were used for further experiments.

**Figure 3:**
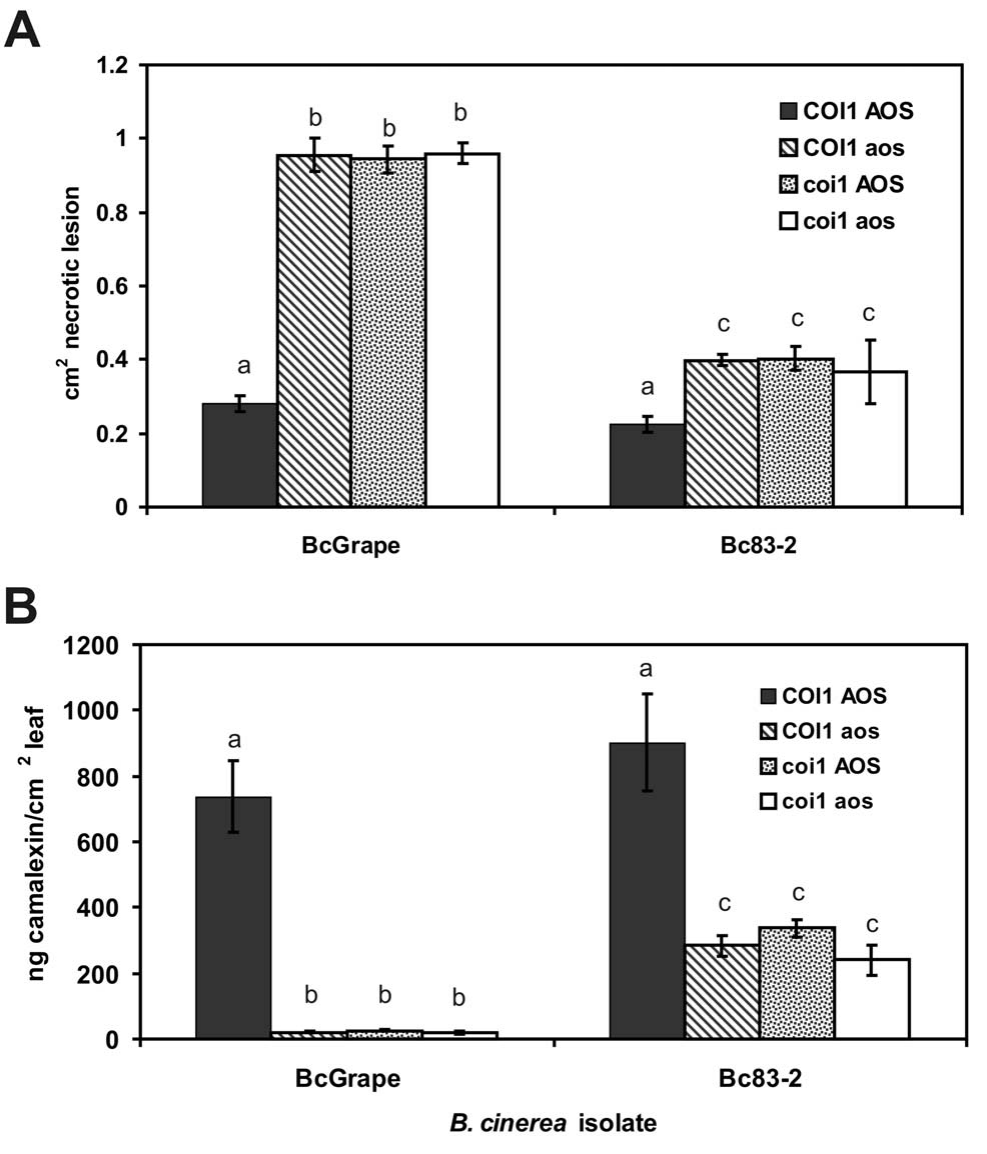
Lesion size and camalexin accumulation in *A. thaliana* deficient in both synthesis and perception of jasmonates. **A**) Mean (± SE) area of necrotic lesions formed by *B. cinerea* isolates BcGrape or Bc83-2 on wild-type, *coi1*, *aos*, and *coi1 aos* double mutant plants at 72 hours post-inoculation **B**) Mean (± SE) camalexin accumulation in wild-type (Col-0), *coi1*, *aos*, and *coi1 aos* double mutants plants infected with *B. cinerea* isolates BcGrape or Bc83-2. Within each figure, letters above bars indicate statistical significance; bars not sharing letters represent significant mean differences at p<0.05.

One hypothesis that could explain the differential accumulation of camalexin in BcGrape and Bc83-2 infected jasmonate-deficient plants is that one of the *B. cinerea* isolates produces a molecule that stimulates the intact jasmonate perception in the *A. thaliana aos* mutant. To determine whether plant deficiencies in jasmonate synthesis and jasmonate perception create similar infection phenotypes and show fully overlapping effects on plant defense signaling, we generated a double mutant containing both *aos* and the *coronatine-insensitive 1* (*coi1*) mutation that confers deficiency in jasmonate perception [42]. A population segregating both *coi1* and *aos* mutations was experimentally infected with BcGrape and Bc83-2. *coi1 aos* double mutant plants displayed infection phenotypes for both tested isolates that did not differ significantly from those observed in either the single mutant *coi1* or *aos* plants (Figure 3). Both the *coi1* and *aos* mutations appear recessive for these phenotypes, as infection phenotypes of plants heterozygous for either or both mutations tested did not differ significantly from homozygous wild-type plants (data not shown). The similarity of *coi1* and *aos* phenotypes suggested that camalexin accumulation in jasmonate-deficient plant genotypes infected with Bc83-2 is not likely mediated by isolate-specific production of a metabolite with jasmonate-like *coi1* dependent activity similar to coronatine [43].

Testing this segregating population also showed that the glabrous (*gl1*) mutation, present in the *aos* mutant background and thus segregating in the *aos*×*coi1* F2 population, had no significant effect on lesion size or camalexin accumulation [44]. We additionally tested a downstream component of the JA pathway, utilizing *JAZ1Δ3* mutant plants. These plants produce a modified version of the JAZ1 protein that confers a dominant jasmonate-insensitive phenotype. The *JAZ1Δ3* mutant plants showed defects in *B. cinerea* mediated camalexin induction similar to *aos* and *coi1* plants, but with a less-dramatic increase in lesion size (Figure S1). These defense responses showed similar *B. cinerea* isolate dependency to that observed in *aos* and *coi1*. Thus, *B. cinerea* isolates vary in their stimulation of signaling networks within *A. thaliana* as demonstrated by the ability of Bc83-2 to induce moderate camalexin levels in the absence of a functional jasmonate signaling pathway (Figure 3).

### The interaction of jasmonate-mediated defense with camalexin biosynthesis

**Figure 4 ppat-1000861-g004:**
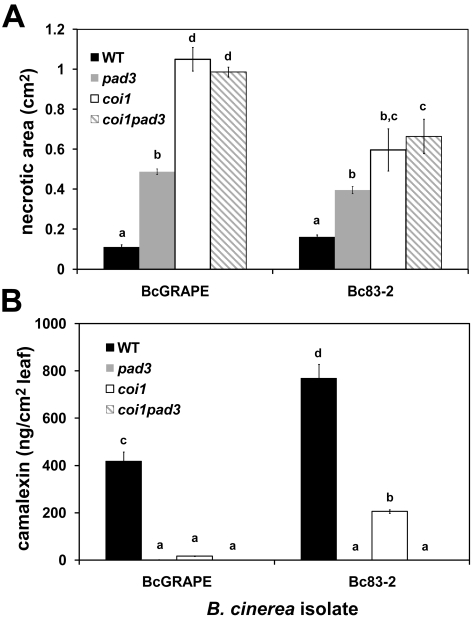
Response to *B. cinerea* infection in jasmonate-insensitive and camalexin-deficient *A. thaliana*. Necrotic area and camalexin accumulation induced by *B. cinerea* isolates BcGrape and Bc83-2 in *A. thaliana* genotypes WT (wild-type Col-0 produced as seed from heterozygous *COI1/coi1*), *pad3*, *coi1*, and *coi1 pad3*. Measurements were taken at 72 hours post inoculation. Within each figure, letters above bars indicate statistical significance; bars not sharing letters represent significant mean differences at p<0.05. **A**) necrotic area (cm^2^ ± SE) **B**) camalexin (ng/cm^2^ leaf area ± SE).

The *A. thaliana Phytoalexin Deficient 3* (*PAD3*) locus encodes a cytochrome P450 enzyme catalyzing the final steps of camalexin biosynthesis [45]. The increased susceptibility of *pad3* mutants to necrotrophic pathogens has supported the conclusion that camalexin is an important defense against these pathogens [28], [46]. We showed that camalexin accumulation depends in part upon an intact jasmonate signaling pathway (Figures 2 and 3). To evaluate the extent that increased susceptibility of jasmonate-insensitive *A. thaliana* genotypes is due to decreased camalexin accumulation in these mutants, we measured development of necrotic lesions and camalexin accumulation in experimentally-infected Col-0 (wild-type), *coi1*, *pad3*, and *coi1 pad3* double mutant plants (Figure 4). Lesion size at 72hpi did not differ between *coi1* and *coi1 pad3* plants, but both of these genotypes developed significantly larger lesions than *pad3* single mutants, indicating that camalexin deficiency explains a significant fraction of, but not the entire increase in, susceptibility of jasmonate mutants to *B. cinerea* (Figure 4A). As anticipated, *pad3* and *coi1 pad3* plants did not accumulate measurable amounts of camalexin (Figure 4B). This observation shows that camalexin accumulation in jasmonate mutants infected with Bc83-2 is not due to a previously-undescribed camalexin biosynthetic capacity in *B. cinerea*. Further, the similarity in lesion size between *pad3* mutant plants infected with BcGrape and Bc83-2 suggests that the difference in susceptibility of jasmonate mutants to these two isolates is not explained by camalexin accumulation in jasmonate mutants infected with Bc83-2.

While BcGrape and Bc83-2 induced similar levels of necrosis on wild-type and *pad3* plants, lesions produced by Bc83-2 on *coi1* and *coi1 pad3* plants were significantly smaller than those produced by BcGrape, supporting our observations that jasmonate deficiency had comparatively less impact on plant susceptibility to Bc83-2 (Figures 2 and 4). Consistent with previous experiments, *coi1* plants infected with BcGrape accumulated extremely low levels of camalexin that did not significantly differ from levels accumulated in *pad3* mutants, and *coi1* plants infected with Bc83-2 accumulated camalexin at levels significantly lower than wild-type but significantly greater than *pad3* mutant plants (Figure 4). In combination, this shows that while camalexin is a large component of the jasmonate-mediated defense against *B. cinerea*, its accumulation does not explain the differential virulence of Bc83-2 and BcGrape on jasmonate-deficient *A. thaliana*.

### Camalexin accumulation in wild-type and jasmonate-insensitive leaves over a time course of *B. cinerea* infection

**Figure 5:**
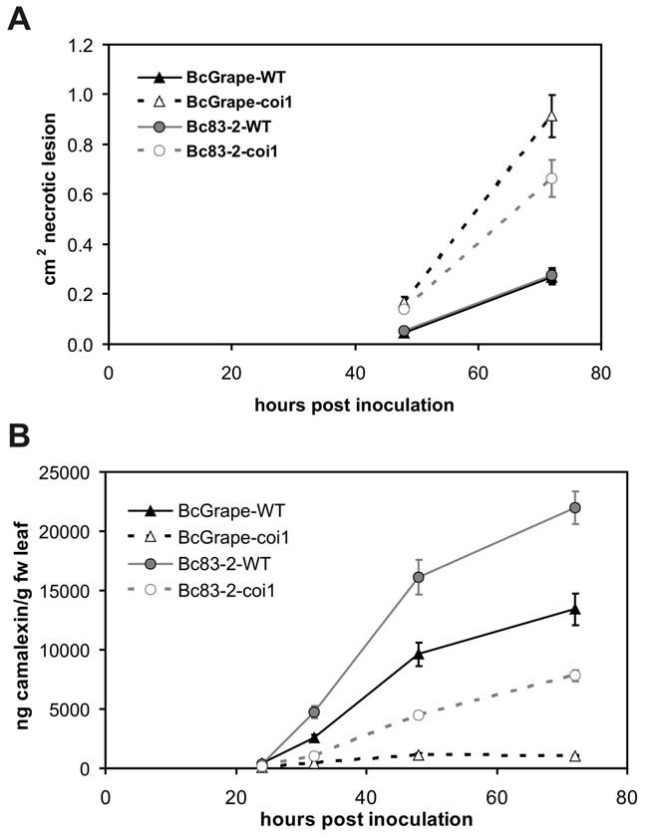
Development of *B. cinerea*-induced necrotic lesions and accumulation of camalexin in wild-type and jasmonate-deficient *A. thaliana* leaves over a four-day infection period. Infection time course for wild-type (solid lines) and *coi1* (dashed lines) *A. thaliana* leaves inoculated with *B. cinerea* isolates BcGrape (triangles) or Bc83-2 (circles), with measurements taken at 24-hour intervals following inoculation. Values presented are the mean (± SE) of three independent time course experiments, with 10 leaves per isolate×genotype×time point within each experiment. Points with non-overlapping error bars represent significant mean differences at p<0.05. **A**) lesion development (cm^2^ necrotic lesion ± SE) **B**) camalexin accumulation (ng camalexin/g leaf tissue ± SE).

To determine whether observed differences in camalexin accumulation and lesion growth between *B. cinerea* treatments were associated with differences in the timing of plant response, time course experiments were conducted using wild-type (*COI1*/*COI1*) and *coi1* mutant plants (Figure 5). *B. cinerea* isolates BcGrape and Bc83-2 produced similarly-sized necrotic lesions on wild-type leaves at 48 hpi, but lesions produced by BcGrape infection of *coi1* leaves rapidly expanded starting at 40–48 hpi. Bc83-2 showed an increase in induced necrosis on *coi1* leaves that was less dramatic than shown by BcGrape but still significantly larger than necroses formed on wild-type leaves. By 32 hpi, camalexin was significantly induced in wild-type but not *coi1* leaves (Figure 5B). Camalexin accumulation at all time points after 24 hpi was highest in wild-type leaves infected with Bc83-2. *coi1* infected with Bc83-2 showed consistently higher levels of camalexin than *coi1* infected with BcGrape. Thus, the difference in camalexin response or virulence between Bc83-2 and BcGrape does not appear to be solely an issue of infection timing but rather variation in pathogen interaction with the plant.

### Transcription of camalexin biosynthetic genes

**Figure 6:**
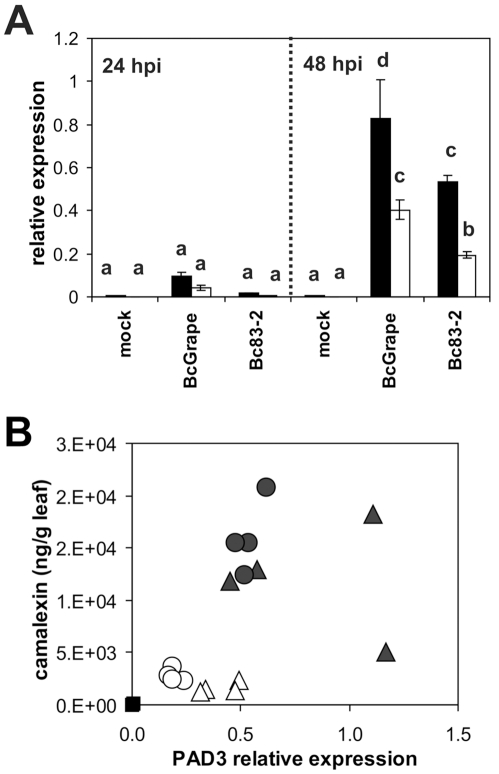
Directed measurement of camalexin biosynthetic transcript as compared to camalexin accumulation in the same tissues. **A**) Relative transcript levels of *PAD3* (At3g26830) in leaves of wild-type (filled bars) and *coi1* (open bars) *A. thaliana* leaves at 24 and 48 hours post-inoculation with *B. cinerea* isolates BcGrape, Bc83-2, or a mock inoculation. Data are means of four independent biological replicates with bars indicating standard error. Within each figure, letters above bars indicate statistical significance; bars not sharing letters represent significant mean differences at p<0.05. **B**) Relative transcript level of PAD3 at 48 hpi (x-axis) in relation to camalexin accumulated (ng/g leaf tissue). Each point represents a single sample. Filled symbols represent samples from wild-type plants and open symbols represent samples from *coi1* plants. Treatments are indicated as: square = mock, triangle = BcGrape, circle = Bc83-2. *PAD3* transcript levels were normalized to transcript levels of reference genes *At4g26410* and *At4g34270* measured in the same samples.

To explore mechanisms controlling altered accumulation of camalexin in jasmonate deficient plants as well as differences between *B. cinerea* treatments, we examined transcript levels of *PAD3* and *CYP71A13*. These genes encode enzymes which catalyze respectively the first committed step and the final steps in camalexin biosynthesis [45], [47], [48]. Relative levels of *PAD3* and *CYP71A13* transcripts were measured at 24 and 48 hours post-inoculation, time points flanking the observed onset of camalexin accumulation (Figure 5B). *PAD3* transcript levels were low but detectable at 24 hours post inoculation (Figure 6A). At 48 hpi, all *B. cinerea* treated samples showed significantly increased *PAD3* transcript accumulation compared to mock treatments. Samples from *coi1* mutants showed less induction of *PAD3* than wild-type samples but the reduction was not commensurate with the observed decrease in metabolite accumulation. While camalexin accumulation was nearly abolished in *coi1* infected with BcGrape, *PAD3* transcript was reduced by only half. Further, Bc83-2 infection is associated with relatively higher camalexin accumulation in *coi1*, but significantly lower *PAD3* transcript accumulation in *coi1* compared to BcGrape infected *coi1*. *CYP71A13* transcript accumulation showed a similar pattern (Figures S2 and S4). Lack of correlation between *PAD3* transcript accumulation and camalexin accumulation measured from the same tissue pool contrasts with previous reports that *PAD3* transcript and camalexin accumulation are highly correlated (Figure 6B) [45]. *B. cinerea* infection with diverse isolates thus reveals evidence of additional regulation of camalexin biosynthesis, beyond transcriptional regulation of known biosynthetic genes.

As camalexin accumulation during *B. cinerea* infection occurs primarily within the plant tissue immediately bordering the developing lesion, it is possible that the spatial distribution of camalexin biosynthetic transcript within an infected leaf may be more relevant to camalexin accumulation than total transcript accumulation within a leaf [30]. To visualize effects of jasmonate insensitivity and *B. cinerea* isolate differences on the pattern of transcript accumulation of the camalexin biosynthetic enzyme *CYP79B2*, we crossed a *CYP79B2* promoter-GUS fusion transgene into a *coi1* background. *CYP79B2* catalyzes the conversion of tryptophan to indole-3-acetaldoxime during camalexin biosynthesis in planta [45], [49]. Leaves from homozygous wild-type and *coi1* plants showing GUS activity were inoculated with *B. cinerea* isolates BcGrape and Bc83-2. Wild-type leaves infected with either *B. cinerea* isolate showed blue staining indicative of GUS activity in a narrow zone bordering the lesion, consistent with previous studies showing that camalexin accumulates primarily within this zone (Figure 1) [30]. *coi1* leaves showed a dramatic difference in staining pattern between BcGrape and Bc83-2 infections, with BcGrape-infected *coi1* leaves showing patterns of GUS activity similar to those seen in wild-type plants, and Bc83-2 infected *coi1* leaves showing intense blue staining within the area visually defined as the necrotic lesion. This intense staining was not associated with increased accumulation of CYP79B2 transcript in *coi1* leaves infected with Bc83-2 (Figure S3). The presence of the *ProCYP79B2:GUS* transgene did not significantly affect camalexin accumulation compared to plants without the transgene from the same segregating F2 population.

**Figure 7:**
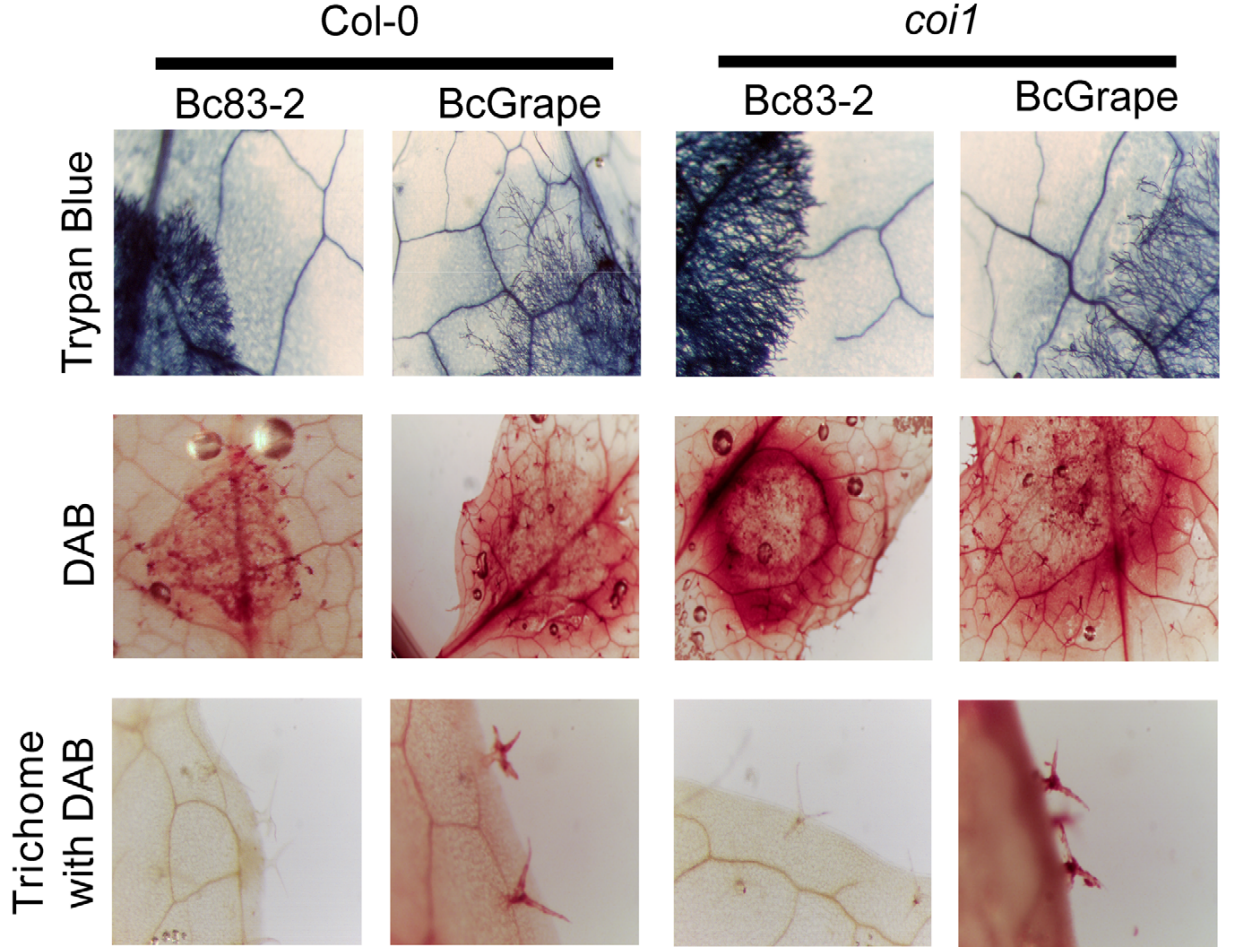
Cellular responses in wild-type and jasmonate-deficient *A. thaliana*. Horizontal labels indicate *B. cinerea* isolate and *A. thaliana* genotypes. Vertical labels indicate the stains applied to the leaves shown; Trypan Blue stains dead plant cells and living fungal hyphae, DAB stains H_2_O_2_ accumulation sites. The bottom row shows H_2_O_2_ accumulation in trichomes located at least 1 cm from the developing necrotic lesion.

A possible explanation for the above observation is that there is less cell death within the Bc83-2 lesion in comparison to BcGrape. We stained infected leaves with a vital stain, Trypan Blue, to compare patterns of cell death associated with infection by the two isolates on wild-type and *coi1* leaves. This showed similarly sized halos of plant cell death surrounding the BcGrape and Bc83-2 lesions on both wild-type and *coi1* leaves that was a lighter color in the *coi1* lesions (Figure 7). Interestingly, these areas contained no detectable fungal cells, suggesting that plant cell death can be caused by mobile plant or fungal signals. No living or dead plant cells were visible within the hyphal mass, suggesting that *B. cinerea* rapidly consumes material in this region and that the observed difference in camalexin accumulation is not due to differential presence of plant cells. These results suggest that the observed GUS staining pattern is caused by persistence of plant-produced protein within the Bc83-2 lesion, rather than active transcription and translation from the plant genome within the Bc83-2 lesion, implying that the absence of a functional jasmonate signaling network alters the ability of Bc83-2 to degrade or disperse proteins. Trypan Blue staining also showed that the two isolates have different growth habits independent of the plant genotypes tested. Bc83-2 hyphae grew at higher density with a well-defined boundary to the hyphal mass, while BcGrape hyphae grew more sparsely with isolated probing hyphae that grow into the surrounding plant issue.

We further compared the infection phenotypes of BcGrape and Bc83-2 using staining for H_2_O_2_ accumulation (DAB). On wild-type *A. thaliana* leaves, infection by either tested *B. cinerea* isolate was associated with diffuse H_2_O_2_ generation within and around the lesion, suggesting that both the plant and fungus generate H_2_O_2_. In contrast, Bc83-2 caused a strong halo of H_2_O_2_ surrounding the developing lesion on *coi1* whereas the BcGrape lesions were associated with a H_2_O_2_ accumulation pattern similar to that observed in wild-type leaves (Figure 7). As generation of reactive oxygen species, including H_2_O_2_, is associated with production of camalexin, the observed pattern of H_2_0_2_ accumulation supports our earlier observation that Bc83-2 induces camalexin via a jasmonate-independent mechanism that is lacking in BcGrape infections. Interestingly, this staining also showed that infection by BcGrape is associated with a systemic accumulation of H_2_O_2_ in trichomes that was independent of plant jasmonate perception and not seen in leaves infected with Bc83-2 (Figure 7). These *B. cinerea* isolates elicit distinct defense responses from plants that include both jasmonate-dependent and jasmonate-independent phenotypes, suggesting both the danger of oversimplifying models of plant—“*B. cinerea*” interaction and the rich potential of intraspecific studies of this pathogen.

### Transcriptional profiling

To identify additional differences in plant transcriptional response to these two *B. cinerea* isolates and build hypotheses regarding the molecular basis of differences in infection phenotype, whole-genome transcriptional profiles of *A. thaliana* leaves inoculated with *B. cinerea* isolates BcGrape or Bc83-2 were compared to each other and to control leaves using both wild-type and jasmonate-insensitive (*coi1*) plants. Based on directed transcript measurements, where induction of camalexin biosynthetic and other defense-associated transcripts was not detected until 48hpi, transcriptional profiling was performed on samples from this 48hpi time point (Figures 6 and S2). Additionally, both *B. cinerea* isolates had initiated lesions by 48hpi, but lesions at this time point, arising from a single inoculation droplet per leaf, occupy only a small portion of the total leaf area and do not show the large differences in lesion size observed on *coi1* leaves at later time points (Figure 5). Estimates of transcript accumulation obtained from arrays were highly consistent with targeted transcript measures obtained via quantitative RT-PCR, with significant Pearson correlation coefficients ranging from 0.76 to 0.91 (Figure S4). Array data are provided as Dataset S1.

### *A. thaliana* transcriptional responses to *B. cinerea* infection

Of 22810 transcripts represented on the arrays, over half (12,999) showed significant effects for the model transcript = genotype + treatment + (genotype × treatment) even after false-discovery adjustments. The majority (11,989) of these statistically significant transcript changes were associated with treatment where most of these transcripts differed between *B. cinerea*-infected and control leaves, rather than between leaves infected with the two *B. cinerea* isolates. We therefore describe statistically significant plant responses consistent between both pathogen isolates as responsive to “*B. cinerea*”. Transcript accumulation from 1458 genes of the *B. cinerea-*responsive loci identified above showed greater than 2-fold increase in response to *B. cinerea* infection, while transcripts from 1602 genes showed more than 2-fold decrease relative to control samples. Differences in transcript abundance between wild-type and *coi1* plants as well as between *B. cinerea*-inoculated and control plants showed overlap with previous studies [50], [51].

All known enzymes of the camalexin biosynthetic pathway were upregulated by *B. cinerea* infection, with *CYP71A13* and *PAD3* respectively showing 124-fold and 67-fold increases in *B. cinerea* infected leaves. An additional five transcripts contributing to biosynthesis of the camalexin precursor, tryptophan, were also upregulated in response to *B. cinerea*, but less dramatically than camalexin biosynthetic genes (Table S1). Other transcripts showing greater than 2-fold transcriptional effects of *B. cinerea* infection that have been previously identified as contributing to plant defense against fungal pathogens included a camalexin regulator (*PAD4*), the MYB transcription factor botrytis-susceptible 1 (*BOS1*), phenylalanine ammonia lyase (*PAL1*), polygalacturonase-inhibiting protein (*PGIP1*), and pathogenesis response proteins (*PR1*, *PR4*, and *PR5*) (Table S1). Transcripts of *PDF1.2a* and *VSP2*, considered markers for jasmonate signaling, were detected only at extremely low levels in both *B. cinerea*-infected and control leaves from *coi1* plants, further supporting our conclusion that camalexin accumulation in jasmonate mutants infected with Bc83-2 is not attributable to isolate-specific pathogen-mediated jasmonate signaling independent of *coi1* and *aos* (Figures S2 and S4) [52], [53].

#### Pathway responses

To associate biological activities with the numerous transcriptional changes caused by *B. cinerea* infection, genes showing >2-fold transcriptional changes in *B. cinerea*-infected leaves were grouped by annotated associations with metabolic pathways [54]. In addition to upregulation of camalexin and tryptophan biosynthetic genes, described above, transcripts associated with ascorbate-glutathione metabolism, including 10 glutathione transferases, were strongly upregulated by *B. cinerea* infection (Table S1). Genes associated with lignin biosynthesis and jasmonate synthesis and response, including six genes encoding JAZ proteins, also showed positive transcriptional responses to *B. cinerea* infection. Pathways downregulated in *B. cinerea*-infected leaves primarily control core metabolic functions such as biosynthesis of chlorophyll and starch, but transcripts linked with the biosynthesis of aliphatic glucosinolates, metabolites primarily associated with plant defense against insect herbivores, were an exception to this pattern. Aliphatic glucosinolate-associated transcripts, including three regulatory MYB transcription factors, were strongly decreased in *B. cinerea*-infected leaves (Table S1). This is consistent with previously documented local repression of aliphatic glucosinolate biosynthesis by *B. cinerea* infection [55].

#### Identification of putative response networks

We used the *A. thaliana* co-expression database ATTED-II to investigate patterns of co-expression for genes transcriptionally affected by *B. cinerea* infection that are not currently associated with described metabolic pathways. Microarray data have successfully identified genes controlling *A. thaliana—B. cinerea* interactions [50], [56]–[59]. Among the transcripts lacking prior pathway associations, we identified three groups of co-regulated loci that may represent undescribed *B. cinerea* responsive networks (Table 2). These proposed groups, described below, represent hypothesized contributions of these genes to *A. thaliana—B. cinerea* interaction, requiring experimental validation.

**Table 2:** Co-expressed gene networks highly altered by *B. cinerea* treatment.

AGI	Locus	BcFC	Model	Geno	Treat	IXN
**MATE (co-regulated with At3g23550)**						
At3g23550	MATE efflux family protein	30.2	0.98	0.21*	0.65*	0.11*
At5g61160	anthocyanin 5-aromatic acyltransferase 1	28.2	0.98	0.37*	0.43*	0.18*
At3g49620	dark inducible 11; oxidoreductase	19.5	0.98	0.51*	0.26*	0.21*
At5g44420	PDF1.2a (plant defensin 1.2a)	10.7	0.98	0.52*	0.22*	0.24*
At2g26020	PDF1.2b (plant defensin 1.2b)	11.7	0.99	0.53*	0.24*	0.22*
At3g16530	legume lectin family protein	38.2	0.99	0.03*	0.94*	0.02*
At3g26200	CYP71B22	8.8	0.99	0.46*	0.32*	0.21*
At3g55970	oxidoreductase, 2OG-Fe(II) oxygenase	4.4	1.00	0.82*	0.09*	0.09*
At1g10700	ribose-phosphate pyrophosphokinase 3	3.5	0.96	0.25*	0.65*	0.06*
At2g39030	GCN5-related N-acetyltransferase	2.4	0.98	0.92*	0.02*	0.04*
At2g41180	sigA-binding protein-related	2.3	0.97	0.25*	0.45*	0.28*
At1g06160	ORA59; ethylene-responsive factor	6.4	0.98	0.55*	0.28*	0.15*
**DETOX (Co-regulated with At2g04050)**						
At2g04050	MATE efflux family protein	2.4	0.84	*0.06*	0.66*	*0.12*
At2g41730	unknown protein	10.8	0.96	*0.00*	0.93*	*0.03*
At2g04070	similar to ATDTX1	6.0	0.97	0.13*	0.74*	0.10*
At2g04040	ATDTX1, multidrug efflux pump	3.6	0.88	0.07*	0.78*	*0.03*
At2g21640	similar to unknown protein	3.3	0.95	0.30*	0.52*	0.13*
At5g51440	23.5 kDa mitochondrial heat shock protein	3.2	0.90	0.07*	0.79*	*0.05*
At1g05680	UDP-glucosyl transferase 74E2	22.8	0.98	0.03*	0.93*	0.02*
At4g37370	CYP81D8	17.5	0.98	0.03*	0.94*	*0.00*
At2g03760	ST (steroid sulfotransferase)	5.0	0.93	*0.01*	0.90*	*0.02*
At3g22370	AOX1A (alternative oxidase 1A)	3.1	0.97	*0.00*	0.94*	*0.02*
**Glucosinolate Catabolism (putative)**						
At2g45570	CYP76C2	23.5	0.99	0.04*	0.94*	0.02*
At4g16690	esterase/lipase/thioesterase family protein	12.3	0.93	*0.01*	0.92*	*0.00*
At3g48580	xyloglucan∶xyloglucosyl transferase	8.3	0.93	0.19*	0.65*	0.09*
At1g79900	ATMBAC2/BAC2	4.1	0.92	*0.00*	0.91*	*0.01*
At5g39520	unknown protein	3.5	0.82	0.14*	0.66*	*0.03*
At1g23550	SRO2 (SIMILAR TO RCD ONE 2)	2.1	0.83	0.14*	0.67*	*0.03*
At5g48180	kelch repeat-containing protein	8.2	0.98	0.02*	0.96*	*0.01*
At1g80160	lactoylglutathione lyase family protein	18.0	0.96	0.06*	0.90*	*0.00*
At3g62590	lipase class 3 family protein	4.5	0.93	0.05*	0.88*	*0.00*

Co-expressed genes were identified using ATTED-II (atted.jp). Networks are identified by the *B. cinerea*-responsive transcript used for database queries (MATE, DETOX), or by hypothesized function (Glucosinolate Catabolism). Genes shown were significantly altered by *B. cinerea* infection with fold-changes >2. ‘AGI’ = locus identifier from the Arabidopsis Genome Initiative (www.arabidopsis.org). ‘BcFC’ is the fold change in transcript measured in *B. cinerea*-infected versus control leaves. ‘Model’ gives the percent of experimental variance explained (R^2^) by an ANOVA model incorporating class variables genotype (‘Geno’: wild-type vs. *coi1*), treatment (‘Treat’: mock, BcGrape, or Bc83-2) and their interaction (IXN). ‘Geno’, ‘Treat’, and ‘IXN’ give the partial variance explained by each model term; these values sum to the model R^2^. Values shown in bold with an asterisk are significant model terms while those in italics represent non-significant model terms.

**Figure 8:**
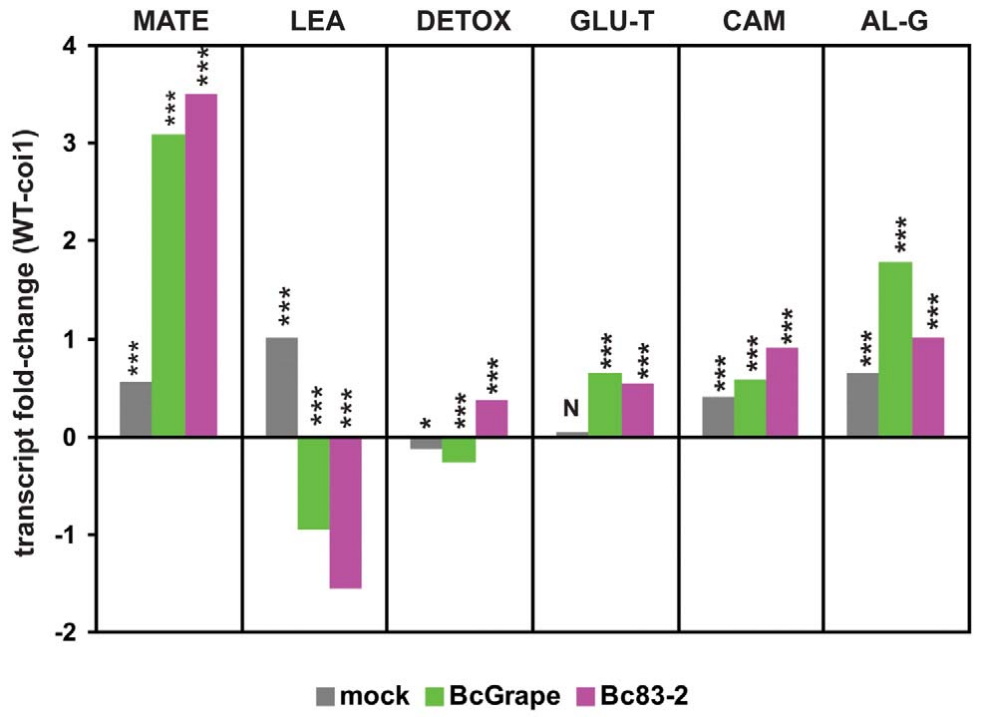
Genotype and *B. cinerea* effects on transcription of co-regulated genes and biosynthetic pathways. The y-axis displays the log_2_ fold-difference in mean expression values between wild-type and *coi1* leaves for groups of transcripts clustered by similarity of expression. Each unit on the vertical axis is equivalent to a 2-fold difference in transcript level. Significant differences between wild-type and *coi1* transcript levels within each treatment are indicated above the bars (‘***’ = p<0.0001, ‘*’ = p<0.05, ‘N’ = p>0.05). MATE (n = 40) and LEA (n = 16) represent groups of coregulated transcripts showing the greatest magnitude of transcript difference between leaves infected with *B. cinerea* isolates BcGrape and Bc83-2 (Table S2). DETOX (n = 16) and GLU-T (n = 15) are transcript clusters upregulated by *B. cinerea* infection. CAM (n = 5) and AL-G (n = 23) are groups of transcripts empirically associated with biosynthesis of camalexin and aliphatic glucosinolates, respectively. Lists of loci associated with these transcripts are provided in Tables 2 and S1.

*DETOX:* Transcript from the *At2g04050* locus, encoding a Multidrug and Toxin Extrusion (MATE) efflux family protein, has been previously documented to increase in response to elevated soil concentrations of boron, tri-nitro toluene, and NaCl [60]–[62]. 16 *B. cinerea* responsive transcripts were identified as co-regulated with *At2g04050* These transcripts were induced by both *B. cinerea* isolates, but accumulated to lower levels in *coi1* leaves infected with BcGrape than wild-type leaves infected with BcGrape, while Bc83-2 infected leaves showed an opposite pattern (Figure 8). This suggests that jasmonate signaling activates this putative network in response to BcGrape but represses it in response to the Bc83-2 isolate, indicating that intraspecific pathogen diversity can affect the outcome of jasmonate signaling. The genes in this network included several multidrug transporters that may act in response to fungal toxins, and *UGT74E2*, a glucosyltransferase implicated in detoxification (Table 2) [60], [63]. This network showed an overrepresentation of ABA response elements (ABRE) suggesting a possible influence of ABA [64], [65].

*Glucosinolate turnover:* Another co-expressed cluster of 15 *B. cinerea*-induced transcripts includes loci encoding enzymes hypothesized to function in catabolism of glucosinolates (Table 2). Glucosinolate turnover may play a role in fungal defense by allowing redistribution of cellular resources stored in glucosinolates to antifungal metabolites [55]. Alternatively, accumulation of these transcripts in response to *B. cinerea* may relate to the function of glucosinolate activation products in pathogen defense and signaling [66], [67]. This hypothesized upregulation of glucosinolate catabolism contrasts with downregulation of transcripts involved in the biosynthesis and activation of aliphatic glucosinolates in *B. cinerea*-infected leaves. Transcripts involved in both the synthesis of aliphatic glucosinolates and their hypothesized catabolism were detected at higher levels in wild-type leaves than *coi1* leaves (Figure 8).

*MATE:* A group of 40 transcripts induced by *B. cinerea* infection were identified by association with the highly *B. cinerea*-responsive locus *At3g23550*, encoding another MATE transporter (Table 2 and Table S2). MATE proteins are associated with resistance to toxins, but may also be involved in transport of plant-produced metabolites required for defense [68]–[70]. This group of transcripts also contains several likely biosynthetic genes, such as acyltransferases, oxidoreductases, and cytochromes P450. Promoter analysis showing an over-representation of two elements, ABRE and GC box, supports coordinated transcriptional response of these genes [64], [65]. Considering inclusion of transcripts previously associated with plant defense against necrotrophic pathogens, such as *PDF1.2* defensins and the ethylene and jasmonate responsive transcription factor *ORA59* (a member of a secondary metabolite regulatory gene family), in this group we hypothesize that these genes contribute to biosynthesis and transport of a currently unknown defense-associated metabolite [71].

### Comparison of transcriptional effects: BcGrape vs. Bc83-2

**Figure 9:**
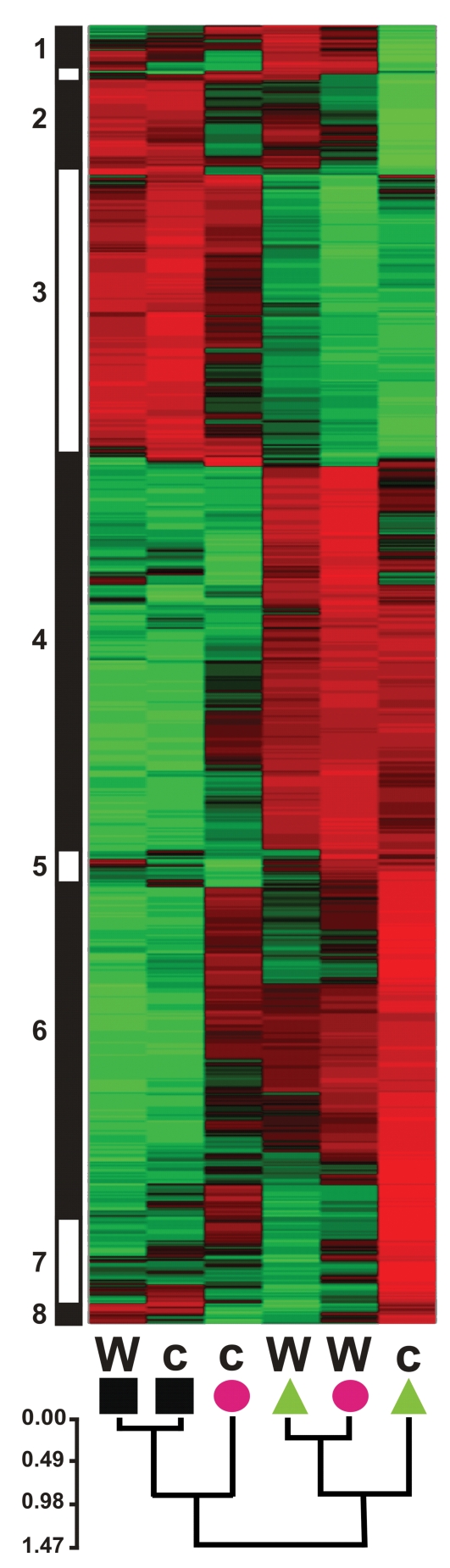
Normalized transcript levels from *A. thaliana* loci showing significant differences in transcript level between *B. cinerea* isolate treatments. Z-score normalized genotype×treatment means for 824 transcripts are clustered vertically by transcriptional similarity among loci using Pearson correlation coefficients and WPGMA. The vertical bar to the left shows rough grouping of transcripts by similarity of normalized expression values; numbers correspond to transcript groups listed in Table S2. Green coloring indicates relatively higher transcript levels; red indicates lower transcription. Horizontal clustering shows similarity among genotype×treatment effects on relative transcript level. Genotype (W = wild-type, c = *coi1*) and treatment (square = mock, triangle = BcGrape, circle = Bc83-2) are indicated at the base of each column. The scale bar shows Pearson correlation distances.

Differences in transcript accumulation after infection by BcGrape or Bc83-2 were generally similar in direction of effect between wild-type and *coi1* leaves but of greater magnitude in *coi1* leaves. Of 824 transcripts showing differential accumulation in response to the two tested *B. cinerea* isolates, 787 show larger differences in *coi1* leaves than wild-type (Table S2). While this correlates with lesion development at later time points, lesion sizes at 48 hours do not significantly differ among genotype×isolate combinations (Figure 5). To identify patterns in these transcript differences that might enhance our understanding of the biology of *A. thaliana* response to *B. cinerea*, we clustered these transcripts by similarity of normalized transcript levels. This identified two large groups of transcripts, those showing relative increases in transcript level in response to *B. cinerea* (clusters 1–3) and those relatively decreased in *B. cinerea*-infected leaves (clusters 4–6) (Figure 9, Table S2). Subsequent clustering of transcript profiles for these loci by genotype and treatment suggested that BcGrape and Bc83-2 infections exert similar transcriptional effects on wild-type leaves. This contrasts with a dramatic difference in transcript patterns observed in *coi1* samples, where Bc83-2 infected *coi1* leaves showed transcript patterns similar to mock-inoculated samples while BcGrape infected *coi1* were more transcriptionally similar to infected wild-type samples. This echoes the pattern observed for the putative DETOX network for *A. thaliana* response to *B. cinerea*, and suggests that jasmonate has opposing transcriptional effects on a set of genes in response to these two *B. cinerea* isolates (Figure 8).

#### Differential transcriptional response to BcGrape

In wild-type plants, the transcript with the greatest increase in accumulation in leaves infected with BcGrape relative to Bc83-2 differed only by 1.75-fold. In *coi1* leaves, however, a greater than 10-fold difference was observed between the two *B. cinerea* treatments. This transcript, *At3g23550* from the above MATE network, and associated transcripts (Table 2), showed greater accumulation in leaves infected with BcGrape relative to leaves infected with Bc83-2 with a differential *coi1* dependence between the two isolates (Table S2, Figure 9 (clusters 3, 4, and 6)).

#### Differential transcriptional response to Bc83-2

The locus associated with the largest transcript difference where Bc83-2 infected leaves showed higher transcript levels than BcGrape infected leaves was *At1g52690*, encoding a late embryogenesis abundant (LEA) protein. LEA proteins are associated with seed maturation, but are also suggested to play important roles in stress tolerance in vegetative tissues [72]. This transcript has not been previously described as pathogen-responsive, but is upregulated in response to exogenous application of ABA and osmotic stress (Genevestigator Response Viewer; www.genevestigator.ethz.ch). In Bc83-2 inoculated wild-type leaves, this transcript accumulated to levels 3-fold greater than those observed in BcGrape-infected wild-type leaves, and more than 11-fold greater than observed in BcGrape-infected leaves from *coi1* plants. However, in the initial analysis of *B. cinerea* effects on transcript abundance, *At1g52690* transcript was identified as downregulated by *B. cinerea* infection, suggesting that this difference represents a failure of Bc83-2 infection or associated plant defense response to reduce transcript accumulated from this locus.

16 associated transcripts showed expression patterns similar to *At1g52690*. All of these loci showed higher relative transcript accumulation in Bc83-2 infected leaves compared to those infected with BcGrape (Table S2). These transcripts were also detected at higher levels in *coi1* leaves infected with either *B. cinerea* isolate than similarly-treated wild-type leaves, suggesting that these transcripts are repressed by jasmonate-mediated response to *B. cinerea* infection (Figure 8). The expression pattern displayed by these transcripts in Bc83-2 infected leaves is similar to the pattern observed in uninfected leaves, further supporting the hypothesis that BcGrape infection represses accumulation of these transcripts, but that this does not occur during a similar stage of infection with Bc83-2 (Figure 9). As such, BcGrape and Bc83-2 differ in induction of both positive and negative transcriptional responses *in planta*.

## Discussion

Jasmonate signaling plays a vital role in plant defense against the highly variable necrotrophic fungal pathogen *B. cinerea* but its molecular effects may differ with pathogen diversity. We show that the genetic diversity contained within *B. cinerea* generates quantitative variation in plant response to the pathogen in the absence of jasmonate synthesis or perception. This variation in plant response was most readily observed as differential accumulation of the *A. thaliana* defense metabolite camalexin, which did not directly correspond with changes in associated biosynthetic transcripts. Analyses of *A. thaliana* transcriptional responses to *B. cinerea* isolates BcGrape and Bc83-2 revealed highly similar changes in transcript levels induced by infection of wild-type plants, yet dramatic differences in transcript profiles between *A. thaliana* infected with these two pathogen isolates when jasmonate signaling is impaired by mutation of *COI1*.

### Regulation of camalexin accumulation

#### Jasmonate signaling controls a substantial portion of camalexin accumulation

Camalexin and jasmonate-mediated defenses have been presented as separate elements of *A. thaliana* resistance to necrotrophic pathogens because jasmonate mutants had no detected effect on the accumulation of camalexin biosynthetic transcripts [51], [73]. We show that, in response to *B. cinerea*, camalexin accumulation was substantially decreased by deficiencies in jasmonate signaling. *A. thaliana* mutants deficient in jasmonate synthesis or jasmonate perception showed significantly lower camalexin accumulation than wild-type plants for all 10 *B. cinerea* isolates tested. Observation of similarly dramatic decreases in camalexin accumulation for jasmonate deficient plants treated with the abiotic elicitors AgNO_3_ and acifluorfen suggests that decreased camalexin accumulation observed in response to *B. cinerea* infection in these genotypes is not due to an active pathogen repression of camalexin biosynthesis or accumulation in the absence of jasmonate signaling (Figure 2). The similarity of responses shown by three mutants deficient in distinct aspects of jasmonate signaling, *aos*, *coi1*, and JAZ1Δ3, suggests that decreased camalexin induction represents a requirement for the entire jasmonate pathway. Yet previous study suggested that camalexin accumulation in response to *B. cinerea* infection does not require *MYC2* [74], a transcriptional regulator of jasmonate signaling, which is repressed by physical interaction with JAZ proteins [75]–[77]. This suggests that jasmonate signaling controls *B. cinerea*-induced camalexin production via an unidentified transcription factor that is repressed by JAZ proteins in a manner similar to *MYC2*.

#### Camalexin biosynthesis is regulated at multiple functional levels

Previous studies report accumulation of camalexin biosynthetic transcripts in *coi1* mutant plants at levels greater than or equal to wild-type in response to *B. cinerea* and oligogalacturonides, leading to the conclusion that jasmonate and camalexin responses are not connected [50], [51]. In light of the common assumption that camalexin transcript levels predict the level of metabolite accumulation, the metabolite is infrequently measured. Our data showed a dramatic decrease in camalexin metabolite accumulation in the *coi1* mutant without an equivalent decrease in transcript levels for the first and last enzymatic steps, *PAD3* and *CYP71A13* (Figures 3 and 6). In addition, variance in camalexin-associated transcripts measured in transcriptional profiling experiments was explained primarily by treatment (*B. cinerea* infection), rather than genotype, despite an obvious effect of plant genotype on camalexin accumulation (Table S1, Figures 3-5). Thus, observed accumulation of these camalexin biosynthetic transcripts is not a reliable surrogate for measurement of metabolite accumulation. These results suggest that additional regulation of camalexin biosynthesis exists, either post-transcriptional regulation or transcriptional regulation involving an unidentified pathway intermediate. While an alternative explanation, that *B. cinerea* degrades camalexin, remains plausible, this is not supported by the observation that camalexin accumulation was also lower in jasmonate-deficient leaves treated with abiotic elicitors of camalexin. The relative performance of BcGrape and Bc83-2 on camalexin deficient *pad3* plants suggests that these isolates are not camalexin-insensitive, and camalexin insensitivity documented in *B. cinerea* is associated with export, rather than degradation, of the metabolite [78]. These data support a deficiency in camalexin biosynthesis by the plant, rather than active degradation by the pathogen that functions only in the absence of jasmonate-mediated plant defense (Figure 2). Thus, jasmonate signaling likely plays a complex regulatory role in camalexin synthesis.

### Similarity of response of wild-type *A. thaliana* to distinct *B. cinerea* isolates

Wild-type (Col-0) *A. thaliana* leaves showed similar responses to the two *B. cinerea* isolates used in these experiments (Figures 1 and 3). These included not only visual and biochemical symptoms (leaf necrosis and camalexin accumulation), but also transcriptional responses to infection (Figures 6 and S2). Comparison with an earlier transcriptional profiling dataset revealed that an unnamed *B. cinerea* isolate showed similar effects to this experiment: of 7718 transcripts described as significantly responding to *B. cinerea* treatment, 6465 showed a significant effect of *B. cinerea* treatment in the experiments described here [51]. Of these, 6107 transcripts showed the same directionality of *B. cinerea* effect. Where the effects of the three *B. cinerea* isolates represented in these two datasets disagree, no single isolate appears to be an outlier. This suggests that, while *B. cinerea* isolates elicit different transcriptional responses from wild-type *A. thaliana*, comparison among datasets reveals a consistent transcriptional signature of *B. cinerea* infection.

### Differential *A. thaliana* response to *B. cinerea* isolates in the absence of jasmonate signaling

While infection of wild-type plants with genetically and phenotypically distinct *B. cinerea* isolates elicited very similar plant responses, infection phenotypes displayed by jasmonate-deficient plants indicate that the phenotypic similarity observed in wild-type plants must be produced by different mechanisms. In particular, the isolate Bc83-2 induces camalexin accumulation both via jasmonate signaling and an additional pathway that is either not induced or specifically blocked by BcGrape infection. Examining differences in transcription between *A. thaliana* leaves infected with these *B. cinerea* isolates revealed that transcriptional responses to these isolates differed more dramatically in jasmonate-insensitive *coi1* plants than in the wild-type background (Figure 9, Table S2). This suggests that jasmonates are not only important signaling components but also integrators of signals from diverse pathogen genotypes into consistent plant defense responses.

The visually distinctive lesion phenotype produced by Bc83-2 infection of jasmonate-deficient *A. thaliana* genotypes, coupled with the persistence of plant-produced GUS activity within the lesion produced by Bc83-2 on *coi1* mutants, initially suggested that the mechanisms by which this isolate induces plant death may be jasmonate-dependent (Figure 1). However, vital staining indicated that the *B. cinerea* isolates caused similar patterns of plant cell death in leaves of both wild-type and jasmonate-insensitive plants (Figure 7). Thus, the observed differences in plant transcriptional response to these pathogen isolates are not likely linked to simple differences in the number of living cells in the leaf, but instead result from differences in plant—pathogen communications, potentially including plant perception of pathogen-induced damage and pathogen metabolism of dead plant tissues.

#### Jasmonate deficiency reveals plant differences in transcriptional regulation that suggest hypotheses regarding the mechanisms controlling plant response to *B. cinerea* variation

The *A. thaliana* transcripts showing the greatest magnitude of differential response to infection of jasmonate-insensitive plants by *B. cinerea* isolates BcGrape or Bc83-2 were an extrusion transporter (elevated in BcGrape treatments compared to Bc83-2) and an ABA-responsive transcript (elevated in Bc83-2 treatments compared to BcGrape). An extrusion transporter might function in plant resistance to *B. cinerea*-produced necrotic toxins, for which isolate differences in biosynthetic capacity have been documented [73]–[75]. Analyses of the secondary metabolic output of BcGrape and Bc83-2 may provide evidence of differential production of candidate phytotoxic compounds to guide future study. Alternatively, *At3g23550* may play a role in plant defense processes independent of export of pathogen-produced toxins, as similar plant MATE transporters are implicated in the synthesis and transport of plant-produced compounds such as anthocyanins, nicotine, and salicylic acid [79]–[81].

The ABA-responsive transcript that showed the greatest magnitude of differential transcription favoring Bc83-2 infection, late embryogenesis abundant protein *At1g52690*, also showed an isolate specific interaction consistent with observed differences in lesion development on jasmonate-insensitive plants, where similar transcript levels were observed between BcGrape-infected *coi1* and wild type leaves while Bc83-2 infected *coi1* leaves showed elevated transcript levels in comparison with wild-type. The group of transcripts identified as co-regulated with this gene (LEA) contains a set of genes that are annotated as ABA-responsive and show enrichment for promoter motifs associated with ABA regulation (Table S2). Transcript accumulation from this group of genes was generally decreased in *B. cinerea-*infected wild-type plants (Figure 8). This suggests that ABA signaling contributes to differentiation of these two isolates *in planta*. While ABA antagonism of both salicylate and jasmonate-mediated plant defenses has been described, the observed increase in accumulation of these transcripts in the absence of functional jasmonate signaling suggests that jasmonate signaling also antagonizes ABA [8], [9], [13], [82], [83]. *B. cinerea* as a species can produce ABA, and blocking activation of ABA signaling via use of the competitive inhibitor beta-aminobutyric acid has been shown to increase plant resistance to *B. cinerea* [36], [84]–[86]. Both of these *B. cinerea* isolates are able to produce ABA, but quantitative analysis of ABA biosynthesis by both the plant and the pathogen during the process of plant infection is necessary to determine the contribution of ABA to differences in infection phenotypes observed in these *B. cinerea* isolates in the absence of intact jasmonate signaling.

### Conclusion

Despite similarities in lesion development and transcriptional effects on wild-type plants, the two *B. cinerea* isolates tested in this study, BcGrape and Bc83-2, show differing interactions with plant response networks that are masked by the response of an intact plant jasmonate signaling pathway. These differences are revealed in mutants deficient in jasmonate biosynthesis and several aspects of jasmonate signaling, most strikingly by quantitative differences in camalexin accumulation in jasmonate-deficient *A. thaliana* leaves infected with these pathogen isolates. Examination of transcriptional response to *B. cinerea* infection in plants with impaired jasmonate signaling has revealed the involvement of at least two groups of co-regulated loci not previously associated with plant defense responses. Exploration of the function of these putative networks in *A. thaliana* defense against *B. cinerea* and other pathogens may provide novel insight into mechanisms of plant defense.

## Methods

### Plant materials

*A. thaliana* mutants deficient in jasmonate biosynthesis, *allene oxide synthase* (*aos*), and biosynthesis of camalexin, *phytoalexin deficient 3* (*pad3-1*), were obtained from the Arabidopsis Biological Resource Center (www.biosci.ohio-state.edu/pcmb/Facilities/abrc/abrchome.htm) [41], [45]. All mutant lines were in the Col-0 genetic background, with *aos* mutants additionally containing the visible marker *gl1*. The presence of a mutant *aos* allele was determined by PCR using gene-specific and insert-specific primers [41]. *A. thaliana* segregating the *coronatine insensitive 1* (*coi1-1*) mutation, conferring deficiency in jasmonate perception, was obtained from J. Glazebrook, University of Minnesota [42]. Homozygous *coi1-1* plants were identified using a CAPS marker; a 531bp fragment of *At2g39940* (*COI1*) contains an Xcm1 restriction site that is abolished by the *coi1-1* mutation [42]. Plants with *coi1 aos* double mutant genotypes were generated by fertilizing *aos* plants with pollen from *COI1/coi1* heterozygous plants. F1 progeny were genotyped to select *COI1/coi1-1* heterozygotes; these were allowed to self-pollinate and *B. cinerea* lesion growth and camalexin accumulation phenotypes were determined for a segregating F2 population. *ProCYP79B2:GUS* contains a transgenic fusion of the *CYP79B2* promoter to a β-glucuronidase reporter [87]. *ProCYP79B2:GUS coi1-1* plants were generated by fertilizing male-sterile *coi1-1* flowers with *ProCYP79B2:GUS* pollen, allowing F1 plants to self-pollinate, and selecting appropriate genotypes from the F2 segregants. *A. thaliana* containing the *JAZ1Δ3::GUS* transgene, conferring a dominant jasmonate-insensitive phenotype, was obtained from G. Howe, Michigan State University [88].

### Plant growth conditions

Plants for all experiments were grown in 36-cell flats (approximately 120cm^3^ soil per cell) in a growth chamber at 12h∶12h light∶dark, 22°C, 50–60% RH, and ∼150µE light intensity. Seed was sown on soil (Sunshine Mix #1, Sun Gro Horticulture Ltd., Bellevue WA) and thinned to one plant per cell at three days post-germination. Genotypes compared within an experiment were systematically interspersed within flats. Plants were sub-irrigated twice weekly with deionized water. Experiments were conducted with mature, non-bolting rosette plants at 5–6 weeks post-planting.

### Treatments

Source and reference data for *B. cinerea* isolates used in this study are provided in Table 1. Preliminary experiments compared infection phenotypes of whole rosettes (detached from the root approximately 0.5cm below the soil surface and placed on agar) with observations of detached single leaves; no differences in measured phenotypes were observed (Figure 1). Further experiments used detached rosette leaves, inoculated with *B. cinerea* spores as previously described [89]. Inoculum was freshly prepared for each experiment from concentrated spore stocks stored at −20°C in 25% glycerol. Leaves were inoculated with 5µl droplets of spore suspension (5×10^5^ spores/ml in half-strength filtered organic grape juice) (Santa Cruz Organics, California USA). Digital photographs were analyzed using Image J to measure lesion area [89], [90]. Control leaves (mock) were inoculated with half-strength grape juice. Abiotic elicitors of camalexin were 5mM AgNO_3_ and 10µM acifluorfen (Sigma-Aldrich, St. Louis, MO USA), applied as four 5µl droplets per leaf to one side of the midvein.

Staining of *ProCYP79B2:GUS* leaves for GUS activity at 72 hours post-inoculation was performed as described [91]. Staining of wild-type and *coi1* leaves for cell death (Trypan Blue) and H_2_O_2_ accumulation (DAB) at 72 hours post-inoculation was performed as described [92], [93].

### Camalexin measurements

Camalexin was extracted in 90% MeOH and quantified via HPLC as previously described [30]. Whole leaves were collected in 500µl 90% MeOH in 96- deep-well plates and stored at −20°C until extraction and analysis, except tissue samples used for transcript measurements where fresh tissue was frozen in liquid nitrogen, ground without solvent, and separate aliquots of frozen tissue were removed for RNA isolation and camalexin extraction. Camalexin measurements are standardized by tissue weight (g) or leaf area (cm^2^); leaf weight and area are highly correlated within the *A. thaliana* genotypes used for these experiments.

### Time course experiments

Seed from heterozygous *COI1*/*coi1 A. thaliana* was grown as described (“Plant Growth Conditions”) and genotyped 2–3 days prior to experiments. DNA was isolated from the first true leaves to minimize stress to the plant and maximize leaf tissue available for experiments. Eight leaves were detached from each homozygous wild-type or *coi1* plant, such that each plant contributed one leaf per *B. cinerea* isolate (BcGrape vs. Bc83-2)×time point (24, 32, 48, and 72 hours post-inoculation) combination. At each time point, leaves were photographed and six to eight leaves per plant genotype×*B. cinerea* isolate combination were collected individually into 90% MeOH and processed as described (“Camalexin measurements”).

### Data analysis

Comparisons of lesion and camalexin data for the experiments described above were performed using a 2-way factorial ANOVA model with classes plant genotype and treatment (Table 1). A genotype×treatment interaction term was included in the model. Specific comparisons of least-squares means were evaluated for significance using Tukey's HSD adjusted p-values. Time course experiments were analyzed similarly, but including time point as an additional class variable. These analyses were conducted in SAS (Version 9.1, SAS Systems, Cary NC USA).

### Directed transcript measurements and transcript profiling

#### Plant growth and treatments

Seed from *COI1*/*coi1* heterozygote plants was grown to 5 weeks old as a segregating population. Leaves harvested from plants into trays of 1% phytagar were inoculated with *B. cinerea* isolate Grape, 83-2, or a mock treatment. After inoculation, additional tissue was removed from plants for genotyping. Leaves from homozygous wild-type and *coi1* mutant plants were collected at 24 and 48 hours post-inoculation. Five to seven leaves were pooled for each sample, with each genotype×treatment×time point combination represented by four independent samples. Leaves were collected in 15ml tubes, frozen in liquid nitrogen, and stored at −80°C. Total RNA was isolated from frozen tissue ground in liquid nitrogen by TRIzol extraction (Life Technologies, Grand Island, NY USA) and further purified using the Qiagen RNeasy kit with on-column DNase treatment (Qiagen Inc, Valencia, CA).

#### Directed transcript measurements

Selected defense-related transcripts were measured both to guide experimental design for array transcript profiling experiments and to provide corroborative measurement of transcripts of particular interest. Isolated mRNA was reverse transcribed using Superscript III (Invitrogen, Carlsbad, California). Quantitative RT-PCR was conducted in 50 µl reactions containing 10 ng cDNA, 1× iQ SYBR Green supermix (Bio-Rad Laboratories, Hercules, CA, USA), and 200 or 250 nM of each primer as previously described [74]. Amplification and analysis of cDNA were as described [94]. We analyzed transcripts encoding camalexin biosynthetic enzymes *PAD3* (*At3g26830*; primer sequences (5′ to 3′) GCAAGAGAACGATGGAGATG and TCTTGTCCCCAAGTGTTGTC) and *CYP71A13* (*At2g30770*, primer sequences TCGGTTGCATCCTTCTCTTC and ATATCGCAGTGTCTCGTTGG), jasmonate-responsive proteins *PDF1.2a* (*At5g44420*) and *VSP2* (*At5g24770*), and wound-responsive transcripts *GST1* (*At1g02930*) and *PR5* (*At1g75040*, primer sequences CGATAAGCCGGAAACTTGTC and AAGTGAAGGTGCTCGTTTCG). Primer sequences used for *PDF1.2a* and *VSP2* were as previously published [74]. The reference genes *At4g34270* and *At4g26410* were used for transcript normalization [95].

Significant differences in the mean relative expression of each target transcript were evaluated using an ANOVA model incorporating GENOTYPE (wild-type versus *coi1*), TREATMENT (mock inoculation, *B. cinerea* isolates BcGrape or Bc83-2), and TIMEPOINT (24 or 48hpi) as class variables and including all interaction terms. Specific comparisons between genotypes for each treatment by time point combination were evaluated using pairwise comparisons of least squares means.

#### Genome-wide transcriptional profiling

Transcript profiling of samples collected at 48hpi was performed using *A. thaliana* ATH1 arrays (Affymetrix, Santa Clara, CA USA) and the same RNA samples used for RT-PCR experiments. Reverse transcription of mRNA, hybridization, washing and scanning of arrays were performed by the UC Berkeley Functional Genomics Laboratory (http://microarrays.berkeley.edu/). Four independent biological replicate samples from each treatment group were separately assayed (four chips per treatment × genotype combination). RMA-corrected and quantile normalized individual probe intensities were summarized by probeset using the median-polish algorithm [96]; data are provided as Dataset S1. All preliminary analyses were conducted using the “affy” package within Bioconductor (www.bioconductor.org) [97].

Summary values for each transcript-associated probeset represented on the ATH1 array were analyzed in R using a generalized linear model procedure with a model including the class variables GENOTYPE and TREATMENT as described for directed transcript analyses, as well as a GENOTYPE×TREATMENT interaction term (http://www.r-project.org/)[98]. *P*-values were estimated from an F-distribution, and adjusted for false discovery due to multiple comparisons using the q-value algorithm within R/QVALUE [99]. A similar analysis excluding transcript values for mock-inoculated samples was performed to explicitly identify transcript differences between the two *B. cinerea* isolate treatments.

Transcripts with full model q-values under 0.001 (equivalent to a false discovery rate of one transcript in 1000) were retained for further analysis. Specific model effects genotype, treatment, and their interaction were then considered significant at a threshold of q≤0.01. RMA median-polish values for transcripts showing a significant treatment effect in the model specifically comparing BcGrape to Bc83-2 infected leaves were normalized using a *z*-score transformation, where the overall mean value for each transcript is subtracted from the mean for each genotype×treatment combination and the result is divided by that transcript's overall standard deviation. Pearson correlation coefficients of these z-score normalized transcripts were used to cluster transcripts by the weighted pair-group method with averaging (WPGMA) (www.bioinf.ebc.ee/EP/EP/EPCLUST/). Gene ontology and annotation descriptions for these transcripts were obtained from The Arabidopsis Information Resource (www.arabidopsis.org). In addition, average per-locus transcript values from leaves infected with BcGrape or Bc83-2 were compared within each plant genotype. Transcripts showing the largest magnitude of difference between BcGrape and Bc83-2 infected leaves within each plant genotype were selected for further exploratory analyses. Lists of genes co-regulated with these transcripts were generated using ATTED-II (atted.jp) [100]. These lists were compared with the list of transcripts showing significant isolate differences to identify previously undescribed gene networks associated with differential plant responses to these *B. cinerea* isolates. Analysis of promoter elements was conducted using the Athena package with elements considered significantly overrepresented at a P value of <0.001 [101].

## Supporting Information

Figure S1 Response to *B. cinerea* infection in JAZ1Δ3 mutants. A) lesion size (mean ±SE) at 72hpi with *B. cinerea* isolates BcGrape or Bc83-2; B) camalexin accumulation (mean ±SE) in leaves treated with mock inoculum, BcGrape, Bc83-2, or 5mM AgNO_3_.
(5.37 MB TIF)

Figure S2 Accumulation of *A. thaliana* transcripts related to defense at 48hpi. Jasmonate response: A) PDF1.2, B) VSP2; Wound response: C) GST1, D) PR5; Camalexin Biosynthesis: E) CYP71A13. Transcript measurements obtained by real-time PCR were normalized to reference transcripts At4g34270 and At4g26410. Mean (±SD) values from 4 biological replicates are presented for wild-type (grey bars) and *coi1* (open bars) leaves inoculated with *B. cinerea* isolates BcGrape, Bc83-2, or a mock treatment. In *coi1* samples, PDF1.2a and VSP2 transcripts were detected at levels too low to display here. Asterisks (*) above bars indicate a significant difference between paired wild-type and *coi1* means at p<0.001.
(1.82 MB TIF)

Figure S3 Accumulation of *CYP79B2* transcript in wild-type and *coi1 A. thaliana* leaves in response to *B. cinerea* infection. Transcript measurements are median-polished RMA values obtained by transcript profiling using ATH1 arrays. Values presented are mean (± SD) for pooled samples of wild-type (Col-0) (filled bars) and *coi1* (open bars) leaves at 48 hours post-treatment with BcGrape, Bc83-2, or a mock inoculum. Significance of specific comparisons between wild-type and *coi1* samples at p<0.05 are indicated above bars (‘*”).
(3.05 MB TIF)

Figure S4 Correlation between array-generated transcript accumulation estimates and directed transcript measures obtained from the same biological samples. Array transcript measures, shown on the vertical axes, are normalized within chips via RMA-median polish; directed transcript measures, shown on the horizontal axes, are normalized within samples relative to reference transcripts At4g26410 and At4g34270. The gene name is given in the lower right corner of each graph. Plant genotypes and treatments are differentiated as follows: filled symbols = wild-type plants, open symbols = *coi1* plants; squares = mock treatment, triangles = BcGrape, circles = Bc83-2.
(6.27 MB TIF)

Table S1 Gene and pathway transcription highly altered by *B. cinerea* treatment. Transcript accumulations of genes shown were significantly altered by *B. cinerea* infection with fold-changes >2. ‘AGI’ = locus identifier from the Arabidopsis Genome Initiative (www.arabidopsis.org). ‘BcFC’ is the fold change in transcript measured in *B. cinerea*-infected versus control leaves. ‘Model’ gives the percent of experimental variance explained (R^2^) by an ANOVA model incorporating class variables genotype (‘Geno’: wild-type vs. *coi1*), treatment (‘Treat’: mock, BcGrape, or Bc83-2) and their interaction (IXN). ‘Geno’, ‘Treat’, and ‘IXN’ give the partial variance explained by each model term; these values sum to the model R^2^. Values shown in bold with an asterisk are significant model terms while those in italics represent non-significant model terms. Genes are grouped according to association with specific biosynthetic pathways or metabolic processes.
(0.16 MB DOC)

Table S2 Genes showing significant differences in transcript level between *B. cinerea* isolate treatments. Loci are identified by AGI number (TAIR; www.arabidopsis.org). ‘Cluster’ indicates groups of transcripts showing similar transcription patterns as evaluated by WPGMA clustering of Pearson correlation coefficients (see Figure 9). ‘coiEFFX’ and ‘BcEFFX’ indicate the direction and statistical significance of effects of plant genotype (wild-type or *coi1*) and treatment (mock or *B. cinerea* inoculation). ‘WT(G-8)’ and ‘coi(G-8)’ provide the difference in log_2_ transcript level between leaves infected with BcGrape and Bc83-2 in wild-type and *coi1* plants, respectively. Association of transcripts with the GO annotation terms defense (DEF), jasmonate signaling or response (JA), ABA response (ABA), wounding (WND), and cell death or senescence (D/S) are shown by stars and colored shading within the associated columns. ‘LEA’ and ‘MATE’ similarly indicate transcripts identified as co-regulated with At1g52690 (late embryogenesis abundant) and At3g23550 (multidrug and toxin efflux), associated with the largest differences in transcription identified between BcGrape and Bc83-2. Gene descriptions are as available from TAIR.
(0.18 MB XLS)

Dataset S1 This dataset provides processed transcript accumulation measurements used for analyses in this manuscript. ‘Probeset’ corresponds to the Affymetrix ATH1 array element. Samples are labeled by *A. thaliana* genotype and treatment, with the following coding: ‘WT’ = wild-type Col-0, ‘coi’ = homozygous coi1 mutant; ‘c’ = control/mock inoculation, ‘G’ = *B. cinerea* isolate Grape (BcGrape), ‘R’ = *B. cinerea* isolate 83-2 (Bc83-2).
(5.16 MB TXT)
