# Supplementary figures and images for: Deficiencies in Jasmonate-Mediated Plant Defense Reveal Quantitative Variation in Botrytis cinerea Pathogenesis
Source: PLoS Pathog. 2010 Apr 15;6(4):e1000861. doi: 10.1371/journal.ppat.1000861 (PMC2855333; doi:10.1371/journal.ppat.1000861)

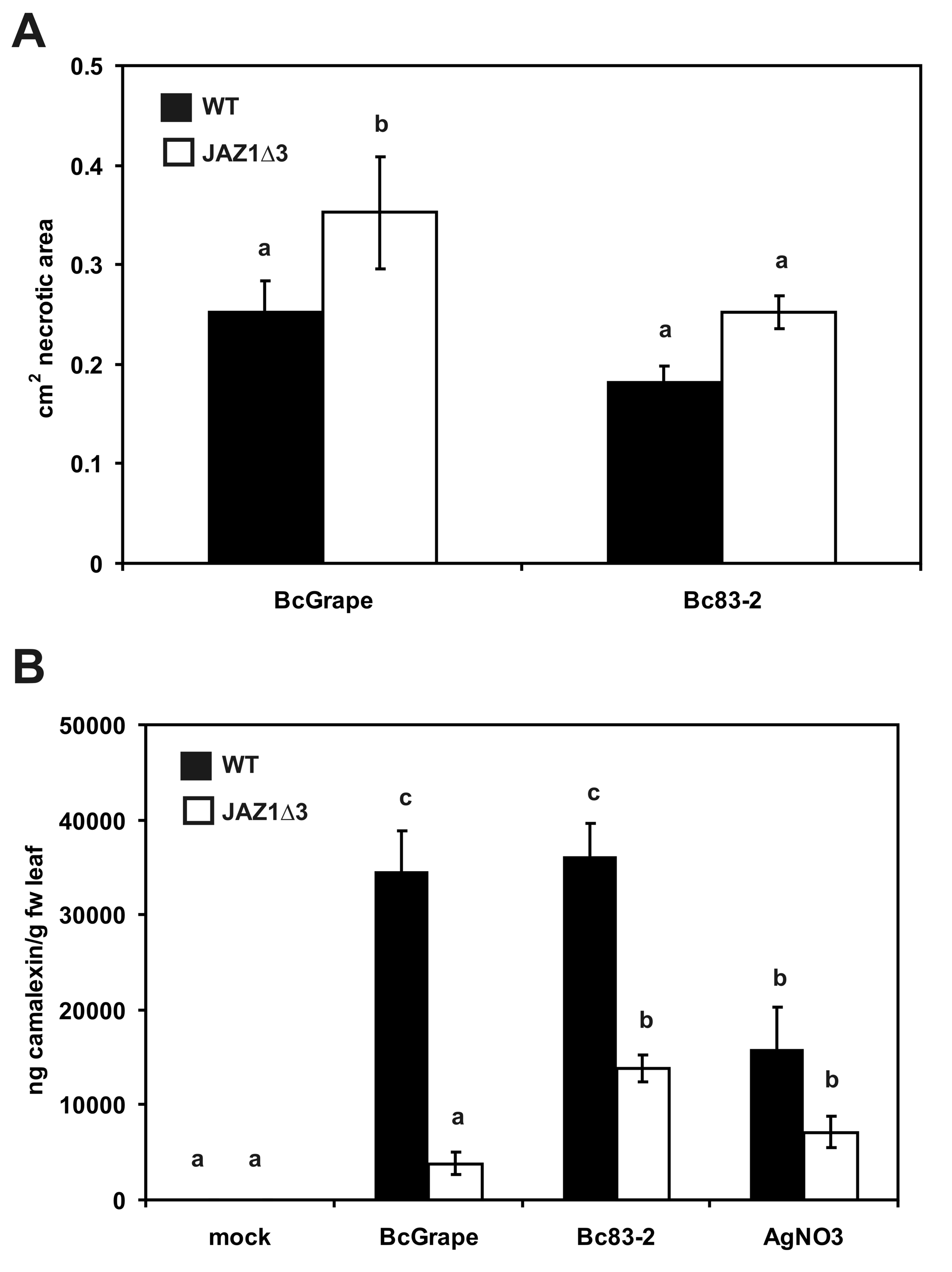

Supplement: Figure S1 — Response to B. cinerea infection in JAZ1Δ3 mutants. A) lesion size (mean ±SE) at 72hpi with B. cinerea isolates BcGrape or Bc83-2; B) camalexin accumulation (mean ±SE) in leaves treated with mock inoculum, BcGrape, Bc83-2, or 5mM AgNO3. (5.37 MB TIF) [file ppat.1000861.s001.tif]

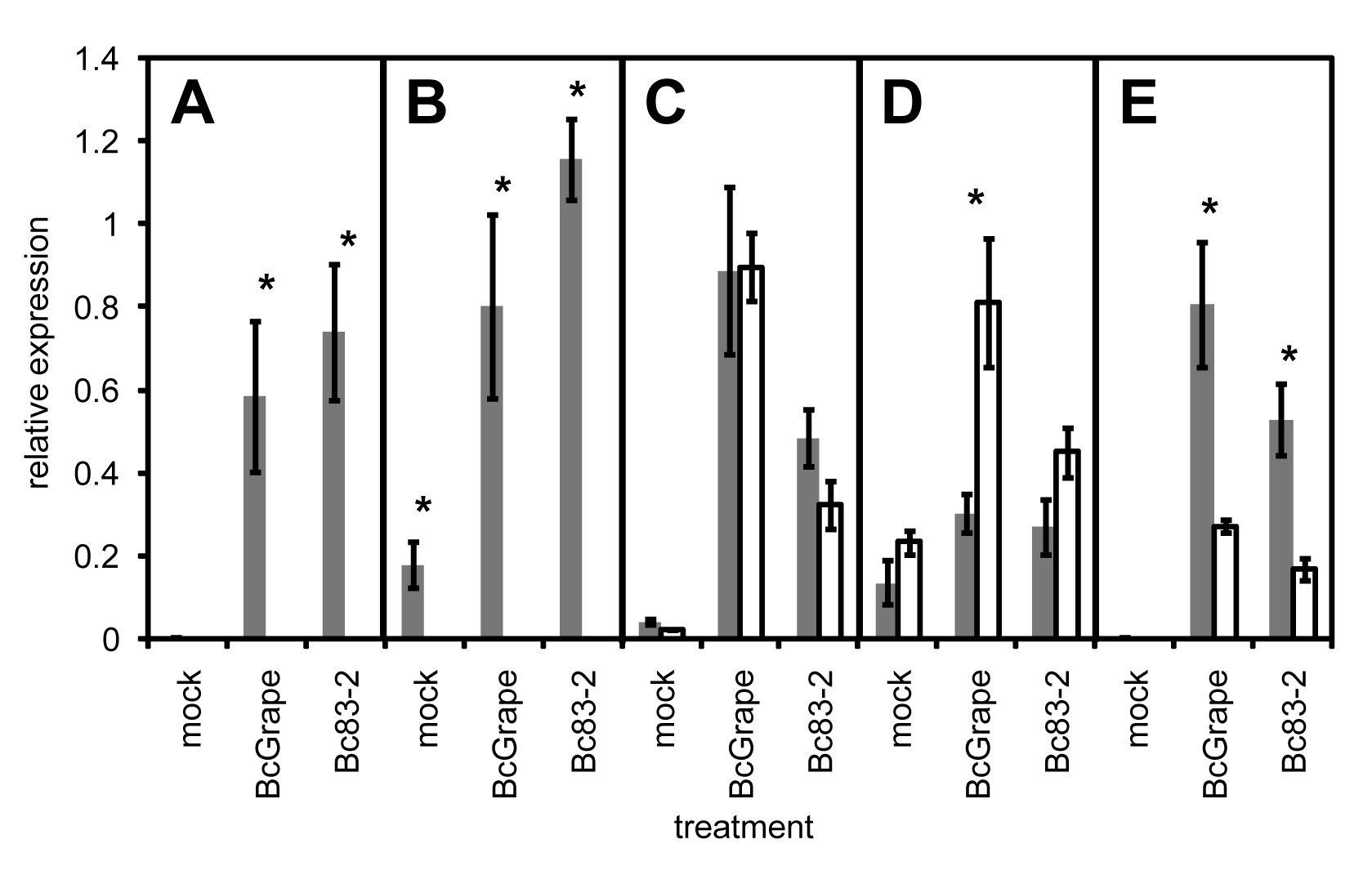

Supplement: Figure S2 — Accumulation of A. thaliana transcripts related to defense at 48hpi. Jasmonate response: A) PDF1.2, B) VSP2; Wound response: C) GST1, D) PR5; Camalexin Biosynthesis: E) CYP71A13. Transcript measurements obtained by real-time PCR were normalized to reference transcripts At4g34270 and At4g26410. Mean (±SD) values from 4 biological replicates are presented for wild-type (grey bars) and coi1 (open bars) leaves inoculated with B. cinerea isolates BcGrape, Bc83-2, or a mock treatment. In coi1 samples, PDF1.2a and VSP2 transcripts were detected at levels too low to display here. Asterisks (*) above bars indicate a significant difference between paired wild-type and coi1 means at p<0.001. (1.82 MB TIF) [file ppat.1000861.s002.tif]

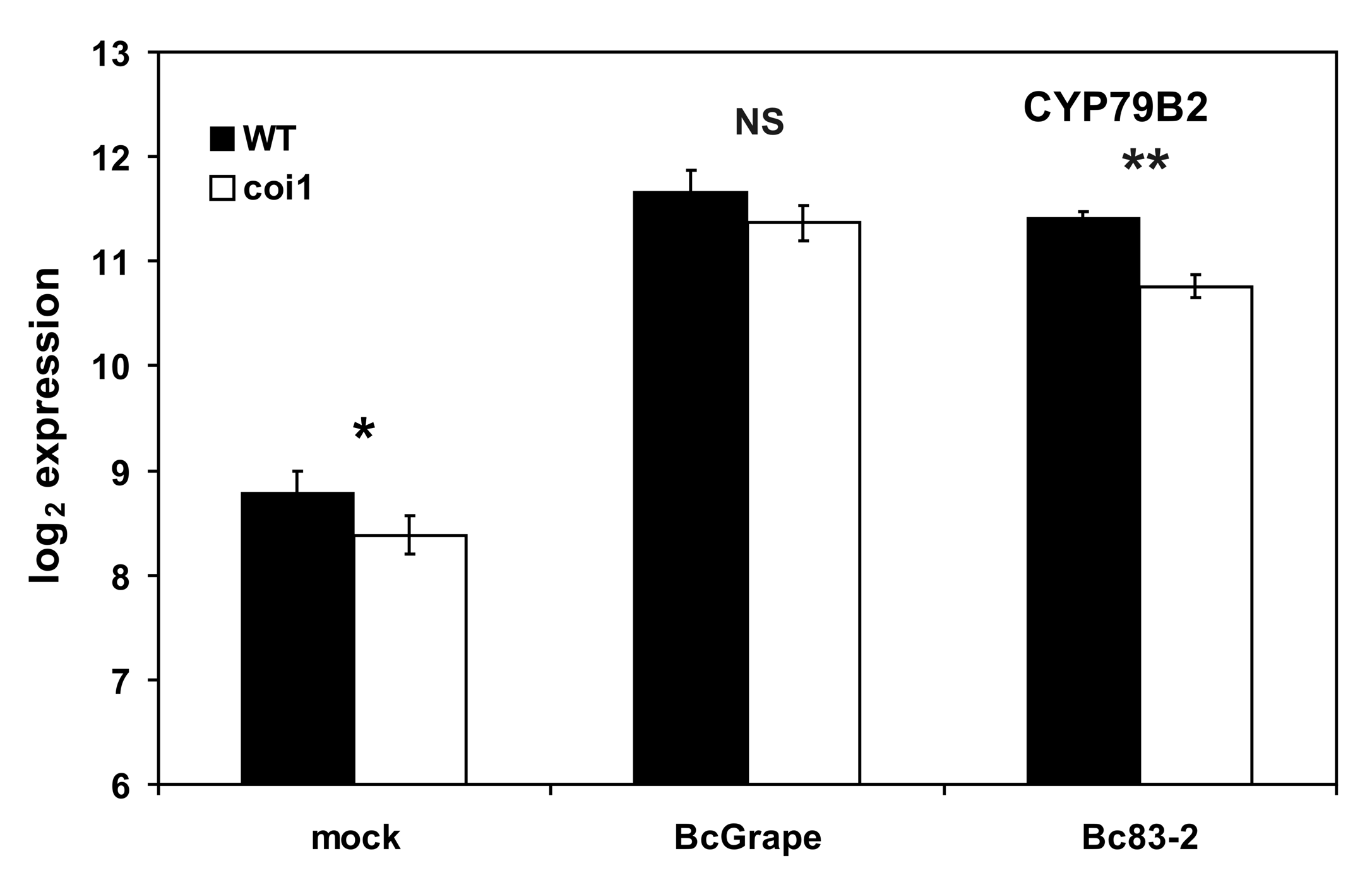

Supplement: Figure S3 — Accumulation of CYP79B2 transcript in wild-type and coi1 A. thaliana leaves in response to B. cinerea infection. Transcript measurements are median-polished RMA values obtained by transcript profiling using ATH1 arrays. Values presented are mean (± SD) for pooled samples of wild-type (Col-0) (filled bars) and coi1 (open bars) leaves at 48 hours post-treatment with BcGrape, Bc83-2, or a mock inoculum. Significance of specific comparisons between wild-type and coi1 samples at p<0.05 are indicated above bars (‘*”). (3.05 MB TIF) [file ppat.1000861.s003.tif]

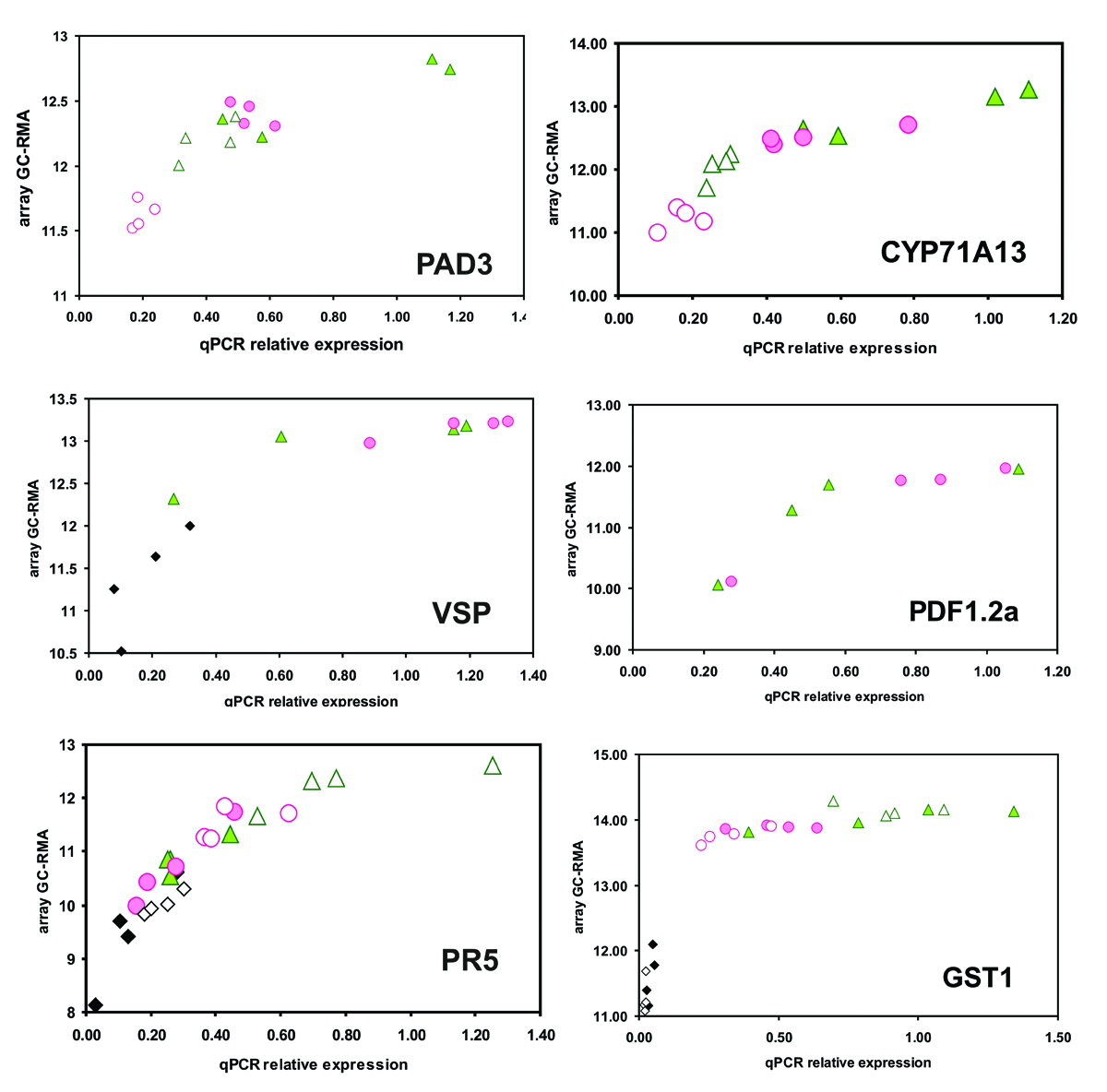

Supplement: Figure S4 — Correlation between array-generated transcript accumulation estimates and directed transcript measures obtained from the same biological samples. Array transcript measures, shown on the vertical axes, are normalized within chips via RMA-median polish; directed transcript measures, shown on the horizontal axes, are normalized within samples relative to reference transcripts At4g26410 and At4g34270. The gene name is given in the lower right corner of each graph. Plant genotypes and treatments are differentiated as follows: filled symbols = wild-type plants, open symbols = coi1 plants; squares = mock treatment, triangles = BcGrape, circles = Bc83-2. (6.27 MB TIF) [file ppat.1000861.s004.tif]
