# Supplementary material for: Deficiencies in Jasmonate-Mediated Plant Defense Reveal Quantitative Variation in Botrytis cinerea Pathogenesis
Source: PLoS Pathog. 2010 Apr 15;6(4):e1000861. doi: 10.1371/journal.ppat.1000861 (PMC2855333; doi:10.1371/journal.ppat.1000861)
Supplement: Table S1 — Gene and pathway transcription highly altered by B. cinerea treatment. Transcript accumulations of genes shown were significantly altered by B. cinerea infection with fold-changes >2. ‘AGI’ = locus identifier from the Arabidopsis Genome Initiative (www.arabidopsis.org). ‘BcFC’ is the fold change in transcript measured in B. cinerea-infected versus control leaves. ‘Model’ gives the percent of experimental variance explained (R2) by an ANOVA model incorporating class variables genotype (‘Geno’: wild-type vs. coi1), treatment (‘Treat’: mock, BcGrape, or Bc83-2) and their interaction (IXN). ‘Geno’, ‘Treat’, and ‘IXN’ give the partial variance explained by each model term; these values sum to the model R2. Values shown in bold with an asterisk are significant model terms while those in italics represent non-significant model terms. Genes are grouped according to association with specific biosynthetic pathways or metabolic processes. (0.16 MB DOC) [file ppat.1000861.s005.doc]

**Table S1: Gene and pathway transcription highly altered by *B. cinerea* treatment.** Transcript accumulations of genes shown were significantly altered by *B. cinerea* infection with fold-changes > 2. ‘AGI’ = locus identifier from the Arabidopsis Genome Initiative ([www.arabidopsis.org](http://www.arabidopsis.org/)). ‘BcFC’ is the fold change in transcript measured in *B. cinerea*-infected versus control leaves. ‘Model’ gives the percent of experimental variance explained (R^2^) by an ANOVA model incorporating class variables genotype (‘Geno’: wild-type vs. *coi1*), treatment (‘Treat’: mock, BcGrape, or Bc83-2) and their interaction (IXN). ‘Geno’, ‘Treat’, and ‘IXN’ give the partial variance explained by each model term; these values sum to the model R^2^. Values shown in bold with an asterisk are significant model terms while those in italics represent non-significant model terms. Genes are grouped according to association with specific biosynthetic pathways or metabolic processes.

| **AGI** | **Locus** | **BcFC** | **Model** | **Geno** | **Treat** | **IXN** |
| --- | --- | --- | --- | --- | --- | --- |
|  |  |  |  |  |  |  |
| **Camalexin Biosynthesis** | |  |  |  |  |  |
| At2g30770 | CYP71A13 | 124.3 | 0.99 | **0.02*** | **0.97*** | *0* |
| At4g39950 | CYP79B2 | 6.6 | 0.96 | **0.03*** | **0.92*** | *0* |
| At3g26830 | phytoalexin deficient 3 | 66.7 | 0.99 | **0.02*** | **0.97*** | *0* |
| At2g30750 | CYP71A12 | 18 | 0.96 | *0.01* | **0.94*** | *0.02* |
|  |  |  |  |  |  |  |
| **Tryptophan Biosynthesis** | |  |  |  |  |  |
| At3g54640 | tryptophan synthase α | 7 | 0.98 | **0.04*** | **0.92*** | **0.03*** |
| At5g05730 | anthranilate synthase α1 | 5.4 | 0.98 | **0.23*** | **0.69*** | **0.06*** |
| At2g04400 | indole-3-glycerol phosphate synthase | 4.7 | 0.98 | **0.05*** | **0.89*** | **0.03*** |
| At4g27070 | tryptophan synthase β | 3.5 | 0.97 | **0.29*** | **0.55*** | **0.13*** |
| At5g17990 | tryptophan biosynthesis 1 | 3.4 | 0.97 | **0.03*** | **0.91*** | *0.03* |
|  |  |  |  |  |  |  |
| **Glutathione Transferases** | |  |  |  |  |  |
| At1g02930 | ATGSTF6 | 5.7 | 0.97 | *0* | **0.97*** | *0.01* |
| At2g29470 | ATGSTU3 | 72.7 | 0.98 | **0.11*** | **0.85*** | **0.02*** |
| At1g17170 | ATGSTU24 | 44.6 | 0.99 | *0* | **0.98*** | *0.01* |
| At1g74590 | ATGSTU10 | 40 | 0.99 | **0.01*** | **0.98*** | *0* |
| At1g17180 | ATGSTU25 | 38.4 | 0.98 | *0* | **0.97*** | *0.01* |
| At2g29460 | ATGSTU4 | 33.1 | 0.99 | **0.08*** | **0.91*** | *0* |
| At1g69930 | ATGSTU11 | 27.8 | 0.98 | *0* | **0.98*** | *0* |
| At1g69920 | ATGSTU12 | 23.1 | 0.96 | **0.12*** | **0.79*** | **0.05*** |
| At2g29490 | ATGSTU1 | 13.3 | 0.98 | **0.14*** | **0.78*** | **0.06*** |
| At2g02930 | ATGSTF3 | 4 | 0.98 | **0.01*** | **0.95*** | *0.02* |
|  |  |  |  |  |  |  |
| **Lignin Biosynthesis** | |  |  |  |  |  |
| At1g67980 | caffeoyl-CoA 3-O-methyltransferase | 22.2 | 0.97 | **0.05*** | **0.91*** | *0.01* |
| At1g76470 | cinnamoyl-CoA reductase | 17 | 0.99 | **0.31*** | **0.56*** | **0.12*** |
| At1g80820 | cinnamoyl CoA reductase 2 | 8.2 | 0.91 | *0* | **0.90*** | *0* |
| At1g72680 | cinnamyl-alcohol dehydrogenase, putative | 3.6 | 0.97 | *0.01* | **0.95*** | *0.01* |
| At1g09500 | cinnamyl-alcohol dehydrogenase family | 26.1 | 0.99 | **0.05*** | **0.92*** | **0.02*** |
| At5g14700 | cinnamoyl-CoA reductase-related | 3.2 | 0.94 | **0.45*** | **0.38*** | **0.10*** |
| At5g19440 | cinnamyl-alcohol dehydrogenase, putative | 2.7 | 0.97 | **0.04*** | **0.90*** | *0.02* |
| At4g34230 | cinnamyl-alcohol dehydrogenase 5 | 2.7 | 0.98 | **0.15*** | **0.78*** | **0.05*** |
| At4g36220 | ferulate-5-hydroxylase 1 | 2.1 | 0.93 | **0.10*** | **0.81*** | *0.03* |
| At4g34050 | caffeoyl-CoA 3-O-methyltransferase | 2 | 0.94 | *0.03* | **0.89*** | *0.02* |
|  |  |  |  |  |  |  |
| **Jasmonate Biosynthesis and Response** | |  |  |  |  |  |
| At3g01420 | α-dioxygenase 1 | 29.4 | 0.97 | **0.21*** | **0.66*** | **0.10*** |
| At1g72520 | lipoxygenase, putative | 11.2 | 0.95 | **0.08*** | **0.87*** | *0.01* |
| At3g25780 | AOC3 (allene oxide cyclase 3) | 8.1 | 0.97 | **0.15*** | **0.80*** | *0.01* |
| At1g17420 | LOX3 (lipoxygenase 3) | 3 | 0.94 | **0.56*** | **0.31*** | **0.07*** |
| At1g55020 | LOX1 (lipoxygenase 1) | 2.5 | 0.97 | **0.58*** | **0.11*** | **0.28*** |
| At2g06050 | OPR3 (OPDA-reductase 3) | 2.3 | 0.99 | **0.68*** | **0.24*** | **0.06*** |
| At1g76690 | OPR2 (12-oxophytodienoate reductase 2) | 2.2 | 0.89 | *0.04* | **0.76*** | *0.1* |
| At1g19180 | JAZ1 | 6.9 | 0.99 | **0.57*** | **0.42*** | *0* |
| At5g13220 | JAZ10, JAS1 | 2.9 | 0.99 | **0.87*** | **0.06*** | **0.05*** |
| At1g17380 | JAZ5 | 3.6 | 0.99 | **0.74*** | **0.15*** | **0.10*** |
| At2g34600 | JAZ7 | 3.1 | 0.99 | **0.62*** | **0.22*** | **0.14*** |
| At1g30135 | JAZ8 | 5.9 | 0.99 | **0.55*** | **0.21*** | **0.22*** |
| At1g70700 | JAZ9 | 2.3 | 0.99 | **0.84*** | **0.08*** | **0.07*** |
| At5g44420 | PDF1.2a (plant defensin 1.2a) | 10.7 | 0.98 | **0.52*** | **0.22*** | **0.24*** |
| At2g26020 | PDF1.2b (plant defensin 1.2b) | 11.7 | 0.99 | **0.53*** | **0.24*** | **0.22*** |
|  |  |  |  |  |  |  |
| **Senescence-Associated** | |  |  |  |  |  |
| At2g35980 | yellow-leaf-specific gene 9 | 90.4 | 0.99 | *0* | **0.99*** | *0* |
| At5g45890 | SAG12; cysteine-type peptidase | 63.4 | 0.9 | *0* | **0.89*** | *0.01* |
| At2g29350 | SAG13; oxidoreductase | 34.3 | 0.98 | **0.12*** | **0.87*** | *0* |
| At2g38860 | yellow-leaf-specific gene 5 | 4.2 | 0.96 | *0* | **0.94*** | *0.02* |
|  |  |  |  |  |  |  |
| **Other genes associated with defense against *B. cinerea*** | |  |  |  |  |  |
| At3g06490 | MYB108 (Botrytis-susceptible 1) | 4.1 | 0.97 | **0.43*** | **0.36*** | **0.18*** |
| At3g52430 | PAD4 (phytoalexin deficient 4) | 2.2 | 0.92 | **0.60*** | **0.25*** | *0.07* |
| At4g23100 | RML1/PAD2; glutamate-cysteine ligase | 2.3 | 0.98 | **0.39*** | **0.45*** | **0.15*** |
| At2g37040 | phenylalanine ammonia lyase 1 | 2.8 | 0.95 | **0.04*** | **0.85*** | **0.06*** |
| At3g53260 | phenylalanine ammonia-lyase 2 | 2.3 | 0.9 | **0.17*** | **0.66*** | *0.07* |
| At5g57220 | CYP81F2 | 21.8 | 0.93 | *0* | **0.92*** | *0* |
| At3g11820 | SYP121 (syntaxin 121); PEN1 | 2.9 | 0.96 | **0.23*** | **0.71*** | *0.02* |
| At5g06860 | polygalacturonase inhibiting protein 1 | 6 | 0.98 | **0.03*** | **0.94*** | *0.01* |
| At2g14610 | PR1 (pathogenesis-related 1) | 10.3 | 0.91 | **0.07*** | **0.81*** | *0.04* |
| At3g04720 | PR4 (pathogenesis-related 4) | 11.9 | 0.97 | **0.16*** | **0.79*** | *0.02* |
| At1g75040 | PR5 (pathogenesis-related 5) | 3 | 0.77 | **0.19*** | **0.55*** | *0.03* |
|  |  |  |  |  |  |  |
| **Aliphatic Glucosinolates** | |  |  |  |  |  |
| At5g23010 | 2-isopropylmalate synthase 3 | -8 | 0.93 | **0.25*** | **0.55*** | **0.13*** |
| At4g03060 | alkenyl hydroxalkyl producing 2 | -8 | 0.96 | **0.15*** | **0.81*** | *0* |
| At3g19710 | branched-chain aminotransferase 4 | -7.6 | 0.97 | **0.39*** | **0.46*** | **0.12*** |
| At5g07690 | myb domain protein 29 | -6.4 | 0.97 | **0.06*** | **0.89*** | *0.02* |
| At5g61420 | myb domain protein 28 | -6.1 | 0.9 | *0* | **0.84*** | *0.06* |
| At4g13770 | CYP83A1 | -5 | 0.94 | **0.20*** | **0.66*** | **0.08*** |
| At4g12030 | bile acid:sodium symporter family protein | -4.7 | 0.94 | **0.31*** | **0.56*** | **0.08*** |
| At3g58990 | aconitase C-terminal domain-containing | -4.5 | 0.94 | **0.38*** | **0.45*** | **0.11*** |
| At1g16410 | CYP79F1 (SUPERSHOOT 1) | -4.3 | 0.95 | **0.38*** | **0.52*** | **0.05*** |
| At2g43100 | aconitase C-terminal domain-containing | -3.9 | 0.91 | **0.26*** | **0.53*** | **0.12*** |
| At1g65860 | flavin-containing monooxygenase family | -3.6 | 0.93 | **0.45*** | **0.45*** | *0.03* |
| At3g03190 | ATGSTF11 | -3.4 | 0.93 | **0.35*** | **0.52*** | **0.06*** |
| At5g23020 | methylthioalkymalate synthase-like | -3 | 0.89 | **0.28*** | **0.47*** | **0.14*** |
| At2g31790 | UDP-glucosyl transferase family protein | -2.6 | 0.88 | **0.22*** | **0.60*** | *0.06* |
| At1g62560 | flavin-containing monooxygenase family | -2.5 | 0.92 | **0.32*** | **0.54*** | *0.06* |
| At5g07700 | myb domain protein 76 | -2.3 | 0.91 | *0.03* | **0.88*** | *0* |
